# Differentiation of Essential Oils Using Nanofluidic Protein Post-Translational Modification Profiling

**DOI:** 10.3390/molecules24132383

**Published:** 2019-06-27

**Authors:** Yasuyo Urasaki, Thuc T. Le

**Affiliations:** College of Pharmacy, Roseman University of Health Sciences, 10530 Discovery Drive, Las Vegas, NV 89135, USA

**Keywords:** capillary isoelectric focusing, *citrus nobilis*, copaifera langsdorffii, *curcuma longa*, JAK/STAT, MAPK, *Melissa officinalis*, nanofluidic protein PTM profiling, pI3K/Akt/mTOR, protein phosphorylation

## Abstract

Current methods for the authentication of essential oils focus on analyzing their chemical composition. This study describes the use of nanofluidic protein post-translational modification (PTM) profiling to differentiate essential oils by analyzing their biochemical effects. Protein PTM profiling was used to measure the effects of four essential oils, copaiba, mandarin, Melissa, and turmeric, on the phosphorylation of MEK1, MEK2, and ERK1/2 in the MAPK signaling pathway; Akt and 4EBP1 in the pI3K/Akt/mTOR signaling pathway; and STAT3 in the JAK/STAT signaling pathway in cultured HepG2 cells. The gain or loss of the phosphorylation of these proteins served as direct read-outs for the positive or negative regulatory effects of essential oils on their respective signaling pathways. Furthermore, protein PTM profiling and GC-MS were employed side-by-side to assess the quality of the essential oils. In general, protein PTM profiling data concurred with GC-MS data on the identification of adulterated mandarin, Melissa, and turmeric essential oils. Most interestingly, protein PTM profiling data identified the differences in biochemical effects between copaiba essential oils, which were indistinguishable with GC-MS data on their chemical composition. Taken together, nanofluidic protein PTM profiling represents a robust method for the assessment of the quality and therapeutic potential of essential oils.

## 1. Introduction

Essential oils are highly concentrated liquids containing complex mixtures of hydrophobic, low molecular weight, and volatile aroma compounds extracted from plants using various extraction methods, including steam distillation, cold pressing, and solvent extraction [1]. The global market for essential oils has increased steadily over the past several decades due to an increasing demand for pure and natural ingredients in various industries, including flavors and fragrances, cosmetics, and aromatherapy [1,2]. Unfortunately, the increased demand for essential oils has also intensified their economically motivated adulteration by unscrupulous providers [3]. In addition to the dishonest economics, adulterated essential oils could pose serious public health consequences, given the proclivity of consumers to use essential oils for their medicinal properties [2,4,5]. 

Adulteration of essential oils includes a broad range of corrupt practices, such as the misrepresentation of botanical species, blending of low-value oils into high-value oils, or the addition of low-cost synthetic materials [1,2]. The level of adulteration of essential oils varies depending on the expertise of the adulterers [6]. The challenges associated with the authentication of essential oils are compounded by the natural variations in chemical composition due to geography, climate, and altitude, and the lack of an international organization that regulates standards for essential oil trade and commerce [3,7,8]. Correspondingly, a broad range of analytical methods are required to determine the authenticity of essential oils, including physical, chemical, chromatographic, spectroscopic, and thermal techniques [9]. Aided by sophisticated chemometrics, such as multivariate analysis and chemical pattern recognition methods with principal component analysis, these analytical methods collectively provide a powerful means for assessing the quality of essential oils [10,11,12,13]. Nonetheless, the authentication of essential oils with existing techniques focuses primarily on their chemical compositions. 

**Figure 1:**
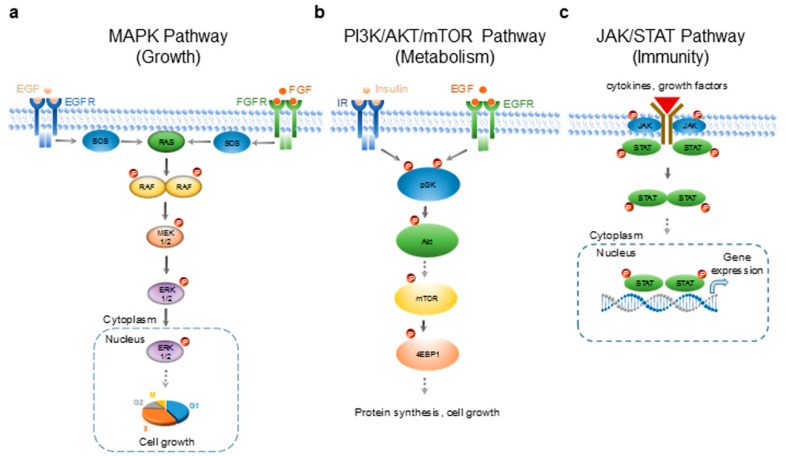
Signaling pathways selected for the evaluation of the biological effects of essential oils. (**a**) The mitogen-activated protein kinase (MAPK) signaling pathway that regulates cell growth and differentiation. EGF—epidermal growth factor; EGFR—epidermal growth factor receptor; FGF—fibroblast growth factor; FGFR—fibroblast growth factor receptor; SOS—guanine nucleotide exchange factors; RAS—small GTPases; serine/threonine-specific protein kinases; MEK1/2—mitogen-activated protein kinase kinase; ERK1/—extracellular signal-regulated kinase 1/2. (**b**) The pI3K/Akt/mTOR signaling pathway that regulates cell metabolism. IR—insulin receptor; pI3K—phosphoinositide 3-kinase; Akt—protein kinase B; mTOR—mammalian target of rapamycin; 4EBP1—eukaryotic translation initiation factor 4E-binding protein 1. (**c**) The JAK/STAT signaling pathway that regulates the cellular immune response. JAK—Janus kinase; STAT—signal transducer and activator of transcription protein. P—phosphate group; solid arrows—direct regulation; dashed arrows—indirect regulation via one or more intermediates. The right to use the DNA double helix illustration was purchased with the standard image license (honglouwawa/shutterstock.com).

In this study, an alternative approach for authenticating essential oils is explored using nanofluidic protein PTM profiling. Rather than focusing on an analysis of the chemical composition, this approach authenticates essential oils by measuring their biochemical effects on selected signaling pathways in cultured HepG2 cells. The HepG2 cells are a human hepatocellular carcinoma cell line that is employed to evaluate the antioxidant, anti-proliferative and hypolipidemic effects of essential oils [14,15]. Essential oils are increasingly being ingested in various liquid forms such as encapsulated in softgel or diluted in drinking water. The first-pass effect suggests that the metabolism of essential oils occurs in the liver or gut. The HepG2 cells serve as an in vitro model to evaluate the effects of essential oils on the signaling activities of liver cells. On the other hand, nanofluidic protein PTM profiling is an automated and high-throughput method that detects protein PTMs using capillary isoelectric focusing (cIEF) immunoassays [16]. It is extremely sensitive to the detection of various protein PTMs, including phosphorylation, acetylation, and glycosylation [17,18]. Previously, nanofluidic protein PTM profiling was applied to detect changes in cellular signaling pathways associated with diseased states or following drug treatments by measuring the percent distribution of protein isoforms in the signaling cascades [19,20,21,22,23,24,25,26,27]. In this study, nanofluidic protein PTM profiling is applied to measure the effects of essential oils on three selected signaling pathways: the MAPK signaling pathway that regulates cell growth and proliferation [28], the PI3K/AKT/mTOR signaling pathway that regulates cell metabolism [29], and the JAK/STAT signaling pathway that regulates the cellular immune response [30] (Figure 1a–c). Essential oils of copaiba, mandarin, turmeric, and Melissa were obtained from different commercial suppliers for side-by-side comparison. This study explores the use of biochemistry as an alternative method to authenticate essential oils. 

## 2. Results and Discussion

### 2.1. Differential Regulation of HepG2 Signaling Pathways by Essential Oils

**Figure 2:**
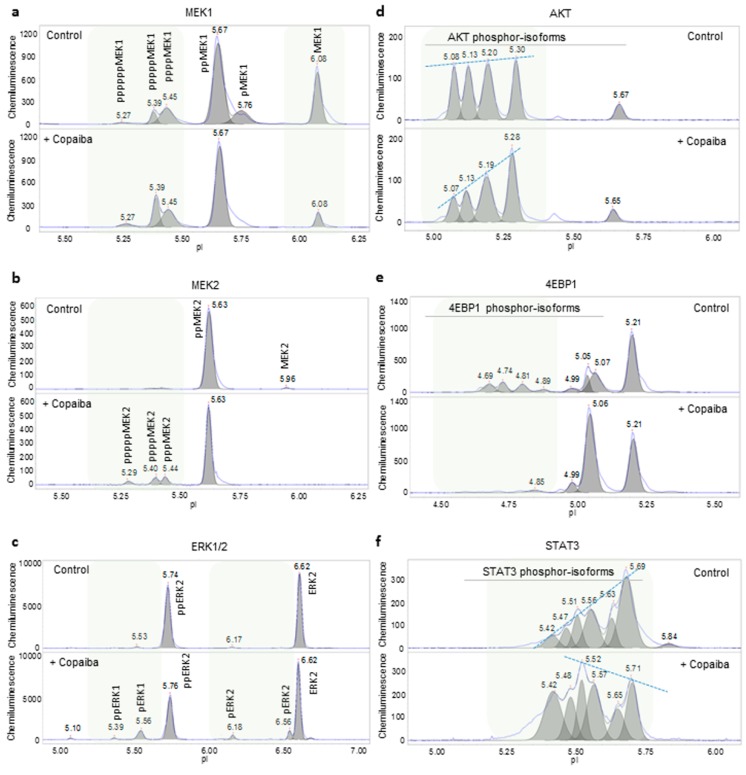
Biological effects of copaiba essential oil on cellular signaling pathways. Copaiba essential oil positively regulated the MAPK signaling pathway by increasing MEK1 phosphorylation (**a**), MEK2 phosphorylation (**b**), and ERK1/2 phosphorylation (**c**). Copaiba essential oil negatively regulated the pI3K/Akt/mTOR signaling pathway by decreasing the levels of phosphorylated Akt (**d**) and 4EBP1 (**e**). Copaiba essential oil positively regulated the JAK/STAT signaling pathway by increasing the level of phosphorylated STAT3 (**f**). Shaded backgrounds highlight areas where substantial changes in the levels of protein isoforms were observed. Dashed lines connect peak intensities to highlight changes in the levels of the phosphorylated Akt and STAT3 isoforms following the treatment of HepG2 cells with copaiba essential oil. CD1 was used for these assessments.

First, nanofluidic protein PTM profiling was applied to examine the effects of essential oils on selected signaling pathways in HepG2 cells. The activity of the MAPK signaling pathway was measured by determining the phosphorylation of several proteins in the signaling cascade, including MEK1, MEK2, ERK1, and ERK2. The treatment of HepG2 cells with copaiba essential oil positively regulated the MAPK signaling pathway by increasing the phosphorylation of MEK1, MEK2, ERK1, and ERK2 (Figure 2a–c and Supplemental Figure S1a–c). The activity of the PI3K/AKT/mTOR signaling pathway was measured by determining the phosphorylation of two proteins in the signaling cascade: AKT and 4EBP1. The treatment of HepG2 cells with copaiba essential oil negatively regulated the PI3K/AKT/mTOR signaling pathway by decreasing the phosphorylation of AKT and 4EBP1 (Figure 2d,e and Supplemental Figure S1d,e). Furthermore, the JAK/STAT signaling pathway was measured by determining the phosphorylation of STAT3. The treatment of HepG2 cells with copaiba essential oil positively regulated the JAK/STAT pathway by increasing the phosphorylation of STAT3 (Figure 2f and Supplemental Figure S1f). Similarly, the effects of green mandarin, Melissa, and turmeric essential oils on these signaling pathways in HepG2 cells were measured using nanofluidic protein PTM profiling and are summarized in Table 1. The effects of copaiba essential oil on the signaling pathways in HepG2 cells were verified with phosphorylation profiling using pathway-specific antibody arrays for the MAPK and pI3K/AKT/mTOR pathways, or nanofluidic protein PTM profiling of additional signaling proteins within the JAK/STAT pathway (Supplemental Figure S2a–e). Clearly, nanofluidic protein PTM profiling was a robust method to rapidly assess the effects of essential oils on cellular signaling pathways. 

### 2.2. Assessment of the Quality of Mandarin and Turmeric Essential Oils 

**Figure 3:**
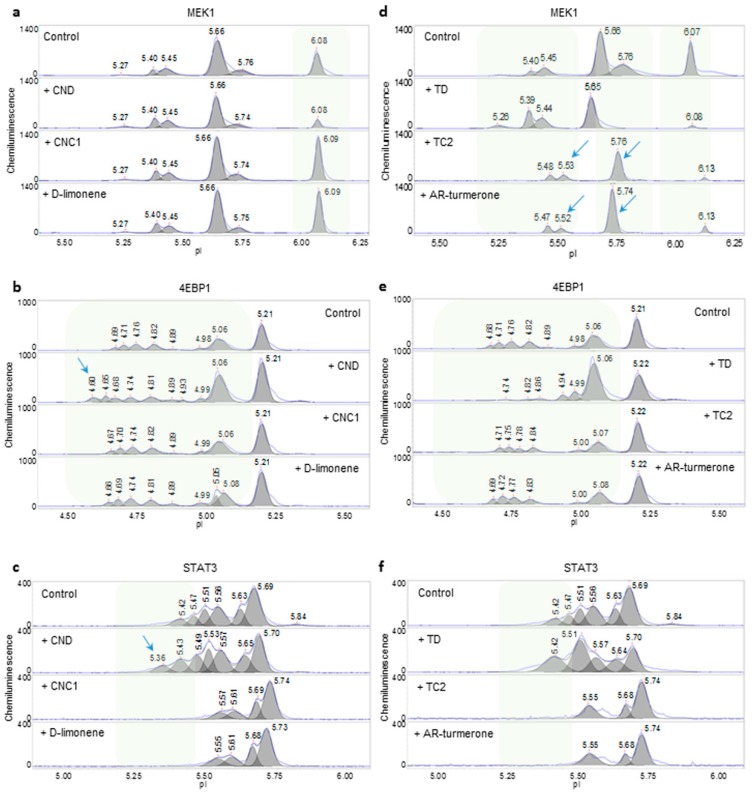
Assessment of the quality of mandarin and turmeric essential oils. (**a–c**) Phosphorylation of MEK1 (**a**), 4EBP1 (**b**), and STAT3 (**c**) observed following the treatment of HepG2 cells with mandarin essential oils CND, CNC1, and D-limonene. (**d–f**) Phosphorylation of MEK1 (**d**), 4EBP1 (**e**), and STAT3 (**f**) observed following the treatment of HepG2 cells with turmeric essential oils TD, TC2, and AR-turmerone. D-limonene and AR-turmerone are the principle components of mandarin and turmeric essential oils, respectively. Shaded backgrounds and blue arrows highlight areas where substantial changes in protein isoforms were observed following the treatment of HepG2 cells with essential oils.

Next, nanofluidic protein PTM profiling was applied to compare the effects of essential oils from different vendors on signaling pathways in HepG2 cells. The phosphorylation profiles of representative signaling proteins MEK1 (MAPK pathway), 4EBP1 (PI3K/AKT/mTOR pathway), and STAT3 (JAK/STAT pathway) are presented in Figure 3a–f. Interestingly, mandarin essential oils from two different vendors exerted different effects on all three signaling pathways. Mandarin essential oil, CND, reduced the level of the unphosphorylated isoform of MEK1, increased the percent distribution of phosphorylated MEK1, and positively regulated the MAPK signaling pathway (Figure 3a). In contrast, mandarin essential oil, CNC1, increased the level of the unphosphorylated isoform of MEK1, reduced the percent distribution of phosphorylated MEK1, and negatively regulated the MAPK signaling pathway. On the other hand, CND induced additional left-shifted peaks for 4EBP1 with low pI values, indicating increased phosphorylation of 4EBP1 and increased activity of the PI3K/AKT/mTOR signaling pathway (Figure 3b). In contrast, CNC1 had no effect on 4EBP1 phosphorylation as compared with the untreated control. Furthermore, CND increased the peak intensity of phosphorylated STAT3 and induced left-shifted peaks for STAT3 with low pI values, which together indicated the positive regulation of the JAK/STAT signaling pathway (Figure 3c). In contrast, CNC1 decreased the levels of phosphorylated STAT3 and negatively regulated the JAK/STAT signaling pathway. Notably, D-limonene, a principle component of mandarin essential oil, mimicked the effects of CNC1 on all three signaling pathways in HepG2 cells.

Turmeric essential oils from different vendors also exerted different effects on signaling pathways in HepG2 cells. For example, turmeric essential oil TD positively regulated the MAPK and JAK/STAT signaling pathways and negatively regulated the PI3K/AKT/mTOR pathway (Figure 3d–f). In contrast, turmeric essential oil TC2 negatively regulated the MAPK and JAK/STAT signaling pathways and had no effect on the PI3K/AKT/mTOR signaling pathway. Interestingly, AR-turmerone, a principle component of turmeric essential oil, mimicked the effects of TC2 on all three signaling pathways in HepG2 cells. 

### 2.3. Assessment of the Consistency of Melissa and Copaiba Essential Oils

**Figure 4:**
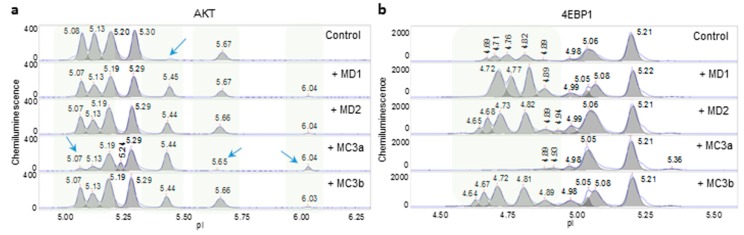
Assessment of the consistency of Melissa essential oils. Levels of phosphorylated Akt (**a**) and 4EBP1 (**b**) isoforms observed following the treatment of HepG2 cells with four Melissa essential oils: MD1, MD2, MC3a, and MC3b. Blue arrows and shaded backgrounds highlight areas where substantial changes in protein isoforms were observed following the treatment of HepG2 cells with Melissa essential oils.

Nanofluidic protein PTM profiling was further applied to assess the consistency of Melissa essential oil. Two Melissa essential oil samples, MD1 and MD2, with different lot numbers consistently induced the appearance of an additional phosphorylated AKT isoform with a pI of 5.45 (Figure 4a). Further downstream of the PI3K/AKT/mTOR signaling pathway, MD1 and MD2 consistently increased the peak intensity of phosphorylated 4EBP1 isoforms (Figure 4b). Both MD1 and MD2 consistently and positively regulated the PI3K/AKT/mTOR signaling pathway. On the other hand, two Melissa essential oils, MC3a and MC3b, exerted different effects on AKT and 4EBP1 phosphorylation. MC3a reduced the levels of phosphorylated AKT with pI values of 5.65, 5.13, and 5.07 and increased the level of the AKT unphosphorylated isoform with a pI of 6.04. In addition, MC3a reduced the levels of the phosphorylated 4EBP1 isoforms. Taken together, MC3a negatively regulated the PI3K/AKT/mTOR signaling pathway. In contrast, MC3b mimicked the effects of MD1 and MD2 on AKT and 4EBP1 phosphorylation and positively regulated the PI3K/AKT/mTOR signaling pathway. 

**Figure 5:**
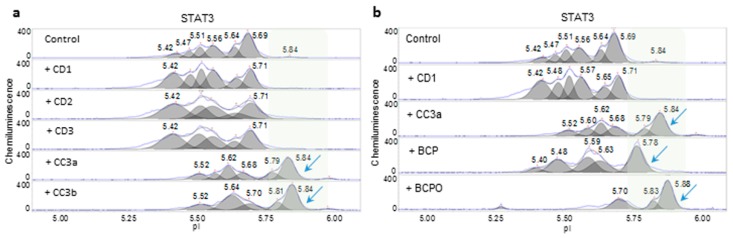
Assessment of the consistency of copaiba essential oils. (**a**) Opposite effects of copaiba essential oils CD1, CD2, and CD3 as compared with CC3a and CC3b on STAT3 phosphorylation. (**b**) β-Caryophyllene and β-caryophyllene oxide caused dephosphorylation of STAT3. Shaded backgrounds and blue arrows highlight right-shifted peaks, which indicate dephosphorylation of STAT3.

An assessment of the consistency of three copaiba essential oil samples from company D (CD1, CD2, and CD3) and two copaiba essential oil samples from company 3 (CC3a and CC3b) was also performed using nanofluidic protein PTM profiling. In general, the effects of copaiba essential oils from company D and company 3 on the MAPK and PI3K/AKT/mTOR signaling pathways were consistent and indistinguishable (Supplemental Figure S3a–c). These similarities were attributed to the presence of β-caryophyllene in all oil samples. β-caryophyllene is a principle component of copaiba essential oil that promoted MAPK signaling and inhibited PI3K/AKT/mTOR signaling (Supplemental Figure S3d–e). Interestingly, the effects of copaiba essential oils from company D on the JAK/STAT signaling pathway were drastically different from those of the oils from company 3. The CD1-3 treatment consistently increased the peak intensity of STAT3 with low pI values, which indicated increased STAT3 phosphorylation and increased activation of the JAK/STAT signaling pathway (Figure 5a). In contrast, CC3a and CC3b treatments consistently caused shifts in STAT3 toward higher pI values and induced the appearance of the unphosphorylated STAT3 isoform around pI 5.84. A β-caryophyllene treatment partially decreased STAT3 phosphorylation but did not completely reproduce the effects of CC3a and CC3b (Figure 5b). On the other hand, β-caryophyllene oxide mimicked the effects of CC3a and CC3b on STAT3 dephosphorylation and induced the appearance of unphosphorylated STAT3 around pI 5.84. 

### 2.4. Analysis of the Chemical Compositions of Essential Oils 

**Figure 6:**
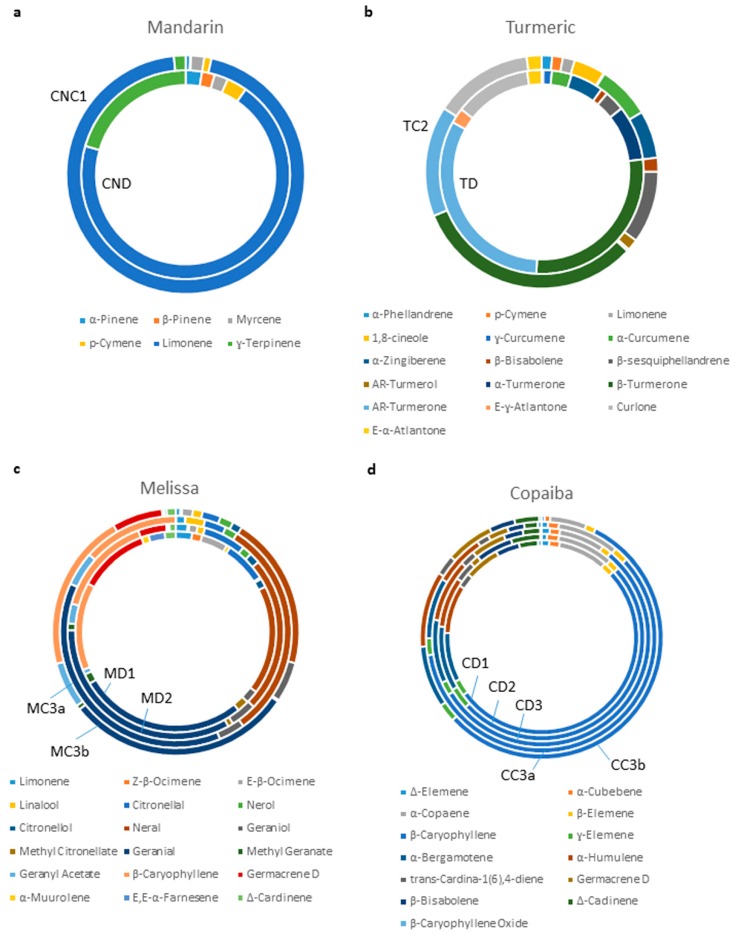
Chemical composition of essential oils. Chemical compositions of selected principle components of (**a**) mandarin essential oils CND and CNC1, (**b**) turmeric essential oils TD and TC2, (**c**) Melissa essential oils MD1, MD2, MC3a, and MC3b, and (**d**) copaiba essential oils CD1, CD2, CD3, CC3a, and CC3b.

The chemical compositions of all essential oils used in this study were analyzed with GC-MS (Supplemental Figures S4–S16 and Supplemental Tables S1–S4). Consistent with the nanofluidic protein PTM profiling data, GC-MS data revealed differences in the chemical compositions of mandarin essential oils CND and CNC1 (Figure 6a, Supplemental Figures S4 and S5 and Supplemental Table S1). CND had a complex mixture of principle components, including D-limonene (65.86%), γ-terpinene (19.43%), para-cymene (2.97%), α-pinene (2.38%) and β-pinene (1.89%). In contrast, the composition of CNC1 was dominated mostly by D-limonene (92.56%), but also contained insignificant quantities of γ-terpinene (1.55%), para-cymene (0.94%), α-pinene (0.60%) and β-pinene (0.12%). Similarly, turmeric essential oils, TD and TC2, exhibited very different chemical compositions (Figure 6b, Supplemental Figures S6 and S7 and Supplemental Table S2). The principle components of TD included AR-turmerone (27.31%), α-turmerone (7.37%), β-sesquiphellandrene (2.75%), and α-curcumene (2.66%). In contrast, the principle components of TC2 included AR-turmerone (13.30%), α-turmerone (0.26%), β-sesquiphellandrene (8.67%), and α-curcumene (6.40%). Furthermore, the inconsistent effect of Melissa essential oil MC3a on the PI3K/AKT/mTOR signaling pathway as compared with the remaining samples MD1, MD2, and MC3b was explained by their chemical compositions (Figure 6c, Supplemental Figures S8–S11 and Supplemental Table S3). MC3a contained substantially elevated levels of the components neral (28.97%) and geranial (35.33%), together with a complete absence of germacrene D (0%). In comparison, the remaining Melissa essential oils had similar ranges of neral (15.30–19.92%), geranial (23.53–28.43%), and germacrene D (3.61–9.88%). Most surprisingly, the chemistry of copaiba essential oils analyzed with GC-MS contradicted the biochemistry analyzed with nanofluidic protein PTM profiling. The GC-MS data revealed that all five copaiba essential oils (CD1, CD2, CD3, CC3a, and CC3b) had nearly identical chemical compositions, including comparable levels of β-caryophyllene and β-caryophyllene oxide (Figure 6d, Supplemental Figures S12–S16 and Supplemental Table S4). However, nanofluidic protein PTM profiling data revealed that CD1, CD2, and CD3 consistently increased, whereas CC3a and CC3b consistently decreased the activity of the JAK/STAT signaling pathway (Figure 5a).

### 2.5. Discussion

Nanofluidic protein PTM profiling represented a robust and rapid biochemical method to authenticate essential oils. In general, the chemical compositions of essential oils were consistent with their biochemical effects on cellular signaling pathways. For example, mandarin essential oil CNC1 had an abnormally high content of D-limonene of 92.56% as compared with the consensus range in the literature of 65.30–77.82% (Figure 6a and Supplemental Table S1). CNC1 also had an abnormally low concentration of γ-terpinene of 1.55% as compared with the consensus range in the literature of 13.13–23.36% [6,31]. In comparison, the chemical constituents of mandarin essential oil CND were present within the expected ranges reported in the literature. Nanofluidic protein PTM profiling revealed that CND positively regulated the MAPK, PI3K/Akt/mTOR, and JAK/STAT signaling pathways. In contrast, CNC1 negatively regulated the MAPK signaling pathway, had no effect on the PI3K/Akt/mTOR signaling pathway, and negatively regulated the JAK/STAT signaling pathway. Both chemical and biochemical analyses concurred that mandarin essential oil CNC1 might have been adulterated. 

The comparison of turmeric essential oils further revealed a consistency between the results of the chemical and biochemical analyses. Turmeric essential oils TD and TC2 exhibited very different chemical compositions with GC-MS analysis. The concentrations of AR-turmerone and α-turmerone in TD were consistent with the literature at 27.31% and 7.37%, respectively (Figure 6b and Supplemental Table S2) [32,33,34]. In comparison, the concentrations of AR-turmerone and α-turmerone in TC2 was abnormally low at 13.30% and 0.26%, respectively. Nanofluidic protein PTM profiling revealed that TD positively regulated the MAPK and JAK/STAT signaling pathways and negatively regulated the PI3K/Akt/mTOR signaling pathway. In contrast, TC2 negatively regulated the MAPK and JAK/STAT signaling pathways and had no effect on the PI3K/Akt/mTOR signaling pathway. Both chemical and biochemical analyses concurred that turmeric essential oil TC2 might have been adulterated. 

The comparison of Melissa essential oils highlighted the capability of the biochemical analysis to detect the consistency of essential oils. Melissa essential oils MD1, MD2, and MC3b had comparable chemical compositions of the major constituents. In comparison, MC3a had up to 89% higher neral and 50% higher geranial concentrations (Figure 6c and Supplemental Table S3). Most significantly, germacrene D was undetectable in MC3a but accounted for up to 10% of the composition of the other three samples. Correspondingly, MD1, MD2, and MC3b consistently increased the activity of the PI3K/Akt/mTOR signaling pathway. In contrast, MC3a decreased the activity of the PI3K/Akt/mTOR signaling pathway. Melissa essential oil is one of the most expensive essential oils due to its low extraction yield, which renders it a frequent target of economically motivated adulteration [10]. The inconsistent chemical composition and biochemical effects of MC3a as compared with the other three oil samples suggested that the MC3a oil sample might have been adulterated. 

Surprisingly, the comparison of copaiba essential oils revealed an inconsistency between the chemical compositions and biochemical effects. All five copaiba essential oils analyzed with GC-MS had nearly identical chemical compositions of the major constituents, including β-caryophyllene, α-copaene, α-bergamotene, and α-humulene, consistent with the reported ranges in the literature [35,36,37] (Figure 6d and Supplemental Table S4). Moreover, the biochemical effects of all five copaiba essential oils on the MAPK and PI3K/Akt/mTOR signaling pathways were identical. Unexpectedly, copaiba essential oils CD1, CD2, and CD3 exerted opposite effects on the JAK/STAT signaling pathway as compared with CC3a and CC3b. Copaiba essential oils CD1, CD2, and CD3 consistently increased the activity of the JAK/STAT signaling pathway. In contrast, copaiba essential oils CC3a and CC3b consistently decreased the activity of the JAK/STAT signaling pathway. Clearly, the biochemical effects on the JAK/STAT signaling pathway differed between the two groups of copaiba essential oils. However, the GC-MS analysis was not able to identify any significant differences in chemical composition among the copaiba essential oils.

Notably, GC-MS analyzes only the volatile components of essential oils. A GC-MS analysis might not be able to detect differences in the quality of essential oils due to the presence of nonvolatile components, impurities, pesticides, stabilizing agents, or other chemicals [9]. In addition, the quality of essential oils might vary without adulteration due to aging, processing, storage, and other factors [38]. Sourcing of essential oils is another important factor for consideration, where essential oils of single origins or multiple origins might have similar chemical compositions in the GC-MS analysis but different biochemical effects and therapeutic potentials [2]. In fact, skilled adulterers are capable of making blends that are indistinguishable from authentic essential oils using a simple GC-MS analysis [6,31]. Broad ranges of sophisticated analytical methods together with chemometrics are necessary to authenticate the chemistry of essential oils [9]. The chemistry responsible for the differences in the biochemical effects of copaiba essential oils was plausibly not detectable with GC-MS. While adulteration that mimics the chemical composition of essential oils has been well documented, adulteration that mimics the biochemical effects of essential oils has not been reported. From this perspective, a nanofluidic protein PTM profiling analysis of the biochemical effects provides a robust complementary approach to methods analyzing the chemical composition for the authentication of essential oils.

In the practice of traditional medicine, whole plant extracts or mixtures of plant extracts are used rather than isolated chemical constituents [39,40]. Emerging scientific evidence obtained in recent years suggests that the biological effects of plant extracts are mediated by the synergy of multiple chemical ingredients rather than single isolated compounds [41,42,43,44,45,46,47]. Interaction of chemical ingredients might: (a) provide synergistic multitargeted effects on biological processes; (b) improve pharmacokinetics with enhanced solubility, resorption rate, and bioavailability; (c) overcome resistance mechanisms of pathogenic microorganisms; and (d) neutralize the adverse side effects of individual compounds [48]. Consistent with the observations from plant extracts, nanofluidic protein PTM profiling data from this study revealed that the biochemical effects of whole essential oils are distinct from the individual constituents. For example, D-limonene and AR-turmerone individually exerted opposite effects to mandarin and turmeric essential oils, respectively, on the MAPK and JAK/STAT signaling pathways. Consistent with the literature, β-caryophyllene oxide negatively regulated the JAK/STAT signaling pathway when administered alone [49,50]. In contrast, whole copaiba essential oils (CD1, CD2, and CD3) positively regulated the JAK/STAT signaling pathway. Previous studies revealed roles for α-bergamotene, β-caryophyllene, and α-humulene in supporting a proper immune response [51,52,53]. Comparable compositions of α-bergamotene, β-caryophyllene, and α-humulene in all five copaiba essential oils were detected using a GC-MS analysis. Nonetheless, CD1, CD2, and CD3 exerted opposite effects on the JAK/STAT signaling pathway as compared with CC3a and CC3b. The differences in the biochemical effects of whole essential oils as compared with their principle constituents might be exploited to assess their authenticity, although further investigation is warranted. In addition to quality assessments, future applications of nanofluidic protein PTM profiling to assess the biochemical effects of essential oils would potentially guide the rational design of their synergistic combinations. 

## 3. Materials and Methods 

### 3.1. Essential Oils 

Copaiba essential oils were obtained from company D (*Copaifera langsdorffii*, CD1, CD2, and CD3) and company 3 (*Copaifera langsdorffii*, CC3a and CC3b). Mandarin essential oils were obtained from company D (*Citrus nobilis*, CND) and company 1 (*Citrus nobilis*, CNC1). Turmeric essential oils were obtained from company D (*Curcuma longa*, TD) and company 2 (*Curcuma longa*, TC2). Melissa essential oils were obtained from company D (*Melissa officinalis*, MD1 and MD2) and company 3 (*Melissa officinalis*, MC3a and MC3b). The names of companies D, 1, 2, and 3 were withheld due to the lack of consent for disclosure and the need to maintain impartiality. All essential oils have different lot numbers. Essential oils of copaiba, Melissa, and turmeric from all vendors were collected with the steam distillation method. On the other hand, Mandarin essential oils from all vendors were collected with the cold pressed method.

### 3.2. Principle Components of Essential Oils 

Principle components of essential oils were purchased from Sigma-Aldrich (St. Louis, MO, USA) and include D-limonene (cat. no. W263303), AR-turmerone (cat. no. 42258), β-caryophyllene (cat. no. W225207), and β-caryophyllene oxide (cat. no. W509647). 

### 3.3. Cell Line 

A human liver cancer cell line, HepG2, was obtained from the American Type Culture Collection (ATCC HB-8065, Manassas, VA) and cultured in ATCC-formulated Eagle’s Minimum Essential Medium supplemented with 10% fetal bovine serum.

### 3.4. Treatment Condition 

The HepG2 cells were cultured to 70% confluence, and then treated with serial dilutions of essential oils ranging from 1:50 to 1:20,000 for 24 h. The effective dilution of essential oils (EC_50_) was determined by the changes in cell density examined using bright-field microscopy. The EC_50_ values for copaiba, green mandarin, Melissa, and turmeric essential oils from company D were 1:200, 1:500, 1:10,000, and 1:1000, respectively. The same dilutions were used for essential oils from other vendors. The EC_50_ values for D-limonene, AR-turmerone, β-caryophyllene, and β-caryophyllene oxide were 37 mM, 46 mM, 25 mM, and 125 µM, respectively. 

### 3.5. Preparation of Total Cell Extracts (TCEs) 

The cultured HepG2 cells (~10^6^ cells) were treated with 60 µl of Bicine/CHAPS lysis buffer (cat. no. 040-764, Protein Simple, Santa Clara, CA) containing proteinase and phosphatase inhibitors and homogenized twice for 6 s. The homogenates were incubated on ice for 10 min, sonicated 4 times for 5 s each, rotated at 4 °C for 2 h, and centrifuged at 12,000 rpm in an Eppendorf 5430R microfuge for 20 min at 4 °C. The supernatants were collected as TCEs for cIEF immunoassays. The TCEs were further prepared in a Premix G2 pH 5–8 separation gradient that contained pI standards (Protein Simple) and transferred to 384 well plates for cIEF immunoassays. 

### 3.6. cIEF Immunoassays 

The cIEF immunoassays were performed using the NanoPro 1000 system (Protein Simple). Samples with a 400 nanoliter volume were separated by isoelectric focusing using the 96-capillary system, followed by immobilization of the proteins on the inner capillary walls using ultraviolet irradiation. Primary antibodies (Supplemental Table S5) and horseradish peroxidase-conjugated secondary antibodies (cat. no. 7074, Cell Signaling, Danvers, MA) were sequentially introduced into the capillaries, followed by chemiluminescence detection reagents. The incubation times were 110 min and 55 min for the primary and secondary antibodies, respectively. The separation time was 50 min at 15,000 microwatts. On average, 40 ng of TCE were loaded into each capillary, and the standard exposure time during signal detection was 240 s. All cIEF immunoassays were performed with a minimum of three repeats for each protein and duplicate experiments per treatment condition, or at least six measurements per protein. High fidelity between repeated measurements was consistent with published reports, with coefficient of variation values ≤0.1 [16]. The Hsp70 was used as a loading control.

### 3.7. Data Analysis 

Assignment of pI values to phosphorylated protein isoforms was based on values published by independent research groups [19,20,21,26,54,55,56,57,58,59], as well as our own published data [17,23,24,25] (Supplemental Table S6). The curve fitting for the area under the curve calculation was performed using Compass software (Protein Simple).

### 3.8. Gas Chromatography Coupled with Mass Spectrometry (GC-MS) Analysis 

The GC-MS analysis of essential oils was performed by an independent third-party lab at the Aromatic Plant Research Center (Lehi, Utah). Briefly, the essential oils were analyzed with a Shimadzu GCMS-QP2010 Ultra operated in the electron impact mode (electron energy of 70 eV) with a scan range of 40–400 atomic mass units and a scan rate of 3.0 scans/second. The GC column was a ZB-5 fused silica capillary column with a 5% phenyl-polymethylsiloxane stationary phase and a film thickness of 0.25 μm. The carrier gas was helium with a column head pressure of 552 kPa and flow rate of 1.37 mL/minute. The injector temperature was 250 °C and the ion source temperature was 200 °C. The GC oven temperature was programmed for an initial temperature of 50 °C, then increased to 260 °C at a rate of 2 °C/minute. A 5% weight/volume solution of the sample in CH_2_Cl_2_ was prepared and 0.1 μL was injected with a splitting mode (30:1). Identification of the oil components was based on their retention indices determined by reference to a homologous series of *n*-alkanes, and by comparison of their mass spectral fragmentation patterns with those reported in the literature and an in-house MS library. 

## Figures and Tables

**Table 1:** Regulation of cellular signaling pathways by essential oils and their principle components.

Essential Oils & Principle Components	MAPK (Growth)	PI3K/AKT/mTOR (Metabolism)	JAK/STAT (Immunity)
Copaiba	+	-	+
Green Mandarin	+	+	+
Melissa	No change	+	No change
Turmeric	+	-	+
β-caryophyllene	+	-	-
D-limonene	-	No change	-
AR-turmerone	-	No change	-

## Supplementary Materials

The following are available online: Figure S1, percentage distribution of protein isoforms following the treatment of HepG2 cells with copaiba essential oil; Figure S2, confirmation of nanofluidic protein PTM profiling data; Figure S3, assessment of the effects of copaiba essential oil and its principle component on the signaling activities of HepG2 cells; Figures S4–S16, chromatograms for essential oils; Figure S4, green mandarin essential oil CND; Figure S5, mandarin essential oil CNC1; Figure S6, turmeric essential oil TD; Figure S7, turmeric essential oil TC2; Figure S8, Melissa essential oil MD1; Figure S9, Melissa essential oil MD2; Figure S10, Melissa essential oil MC3a; Figure S11, Melissa essential oil MC3b; Figure S12, copaiba essential oil CD1; Figure S13, copaiba essential oil CD2; Figure S14, copaiba essential oil CD3; Figure S15, copaiba essential oil CC3a; Figure S16, copaiba essential oil CC3b; Table S1, summary of GC-MS data for mandarin essential oils; Table S2, summary of GC-MS data for turmeric essential oils; Table S3, summary of GC-MS data for Melissa essential oils; Table S4, summary of GC-MS data for copaiba essential oils; Table S5, list of primary antibodies; Table S6, assignment of pI values to protein isoforms.
