# Supplementary material for: Differentiation of Essential Oils Using Nanofluidic Protein Post-Translational Modification Profiling
Source: Molecules. 2019 Jun 27;24(13):2383. doi: 10.3390/molecules24132383 (PMC6651569; doi:10.3390/molecules24132383)
Supplement: Supplementary file 1 [file molecules-24-02383-s001.pdf]

Supplementary Materials for

Differentiation of Essential Oils Using Nanofluidic Protein Post-translational Modification Profiling

Yasuyo Urasaki & Thuc T. Le*

College of Pharmacy, Roseman University of Health Sciences, 10530 Discovery Drive, Las Vegas, NV 89135, USA

*Correspondence and requests for materials should be addressed to T.T.L. (email: tle5@roseman.edu)

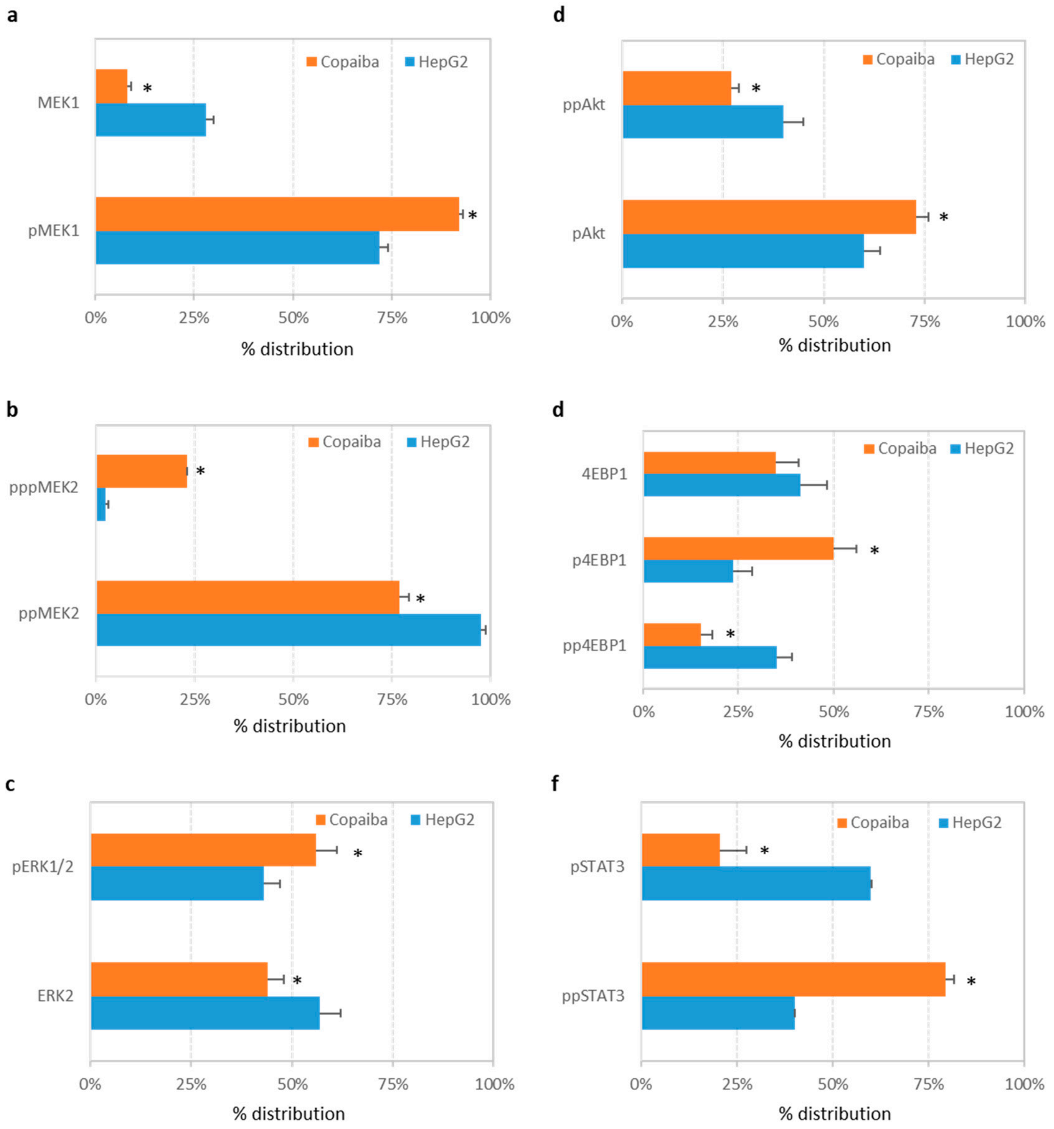

Supplemental Figure S1. Percentage distribution of protein isoforms following the treatment of HepG2 cells with copaiba essential oil. (**a-c**) Consistent increases of phosphor-isoforms of pMEK1 (**a**), pppMEK2 (**b**), and pERK1/2 (**c**) indicated an up-regulation of the MAPK signaling pathway. MEK1: unphosphorylated isoform; pMEK1: phosphorylated isoforms with pI values from 5.76 to 5.25; ppMEK2: MEK2 phosphor-isoform at pI 5.63; pppMEK2: MEK2 phosphor-isoforms with pI values from 5.44 to 5.29; ERK2: unphosphorylated ERK2 isoform with pI 6.63; pERK1/2: all phosphor-isoforms of ERK1 and ERK2. (**d,e**) Consistent decreases of ppAkt (**d**) and pp4EBP1 (**e**) phosphor-isoforms indicated a down-regulation of the PI3K/AKT/mTOR signaling pathway. pAkt: phosphor-isoforms of Akt with pI values from 5.65-5.15; ppAkt: phosphor-isoforms of Akt with pI values from 5.15-5.05. 4EBP1: unphosphorylated 4EBP1 isoform with pI 5.21; p4EBP1: phosphor-isoform of 4EBP1 with pI 5.06; pp4EBP1: phosphor-isoforms of 4EBP1 with pI values from 4.99 – 4.60. (**f**) An increase in ppSTAT3 phosphor-isoforms indicated an up-regulation of the JAK/STAT signaling pathway. pSTAT3: phosphor-isoform of STAT3 with pI 5.69; ppSTAT3: phosphor-isoforms of STAT3 with pI 5.65-5.20. Error bars are standard deviations across six repeated measurements. Asterisks indicate statistical significance with p-value <0.01 against untreated control. p-value was calculated using the paired Student's t-test. CD1 was used for these assessments.

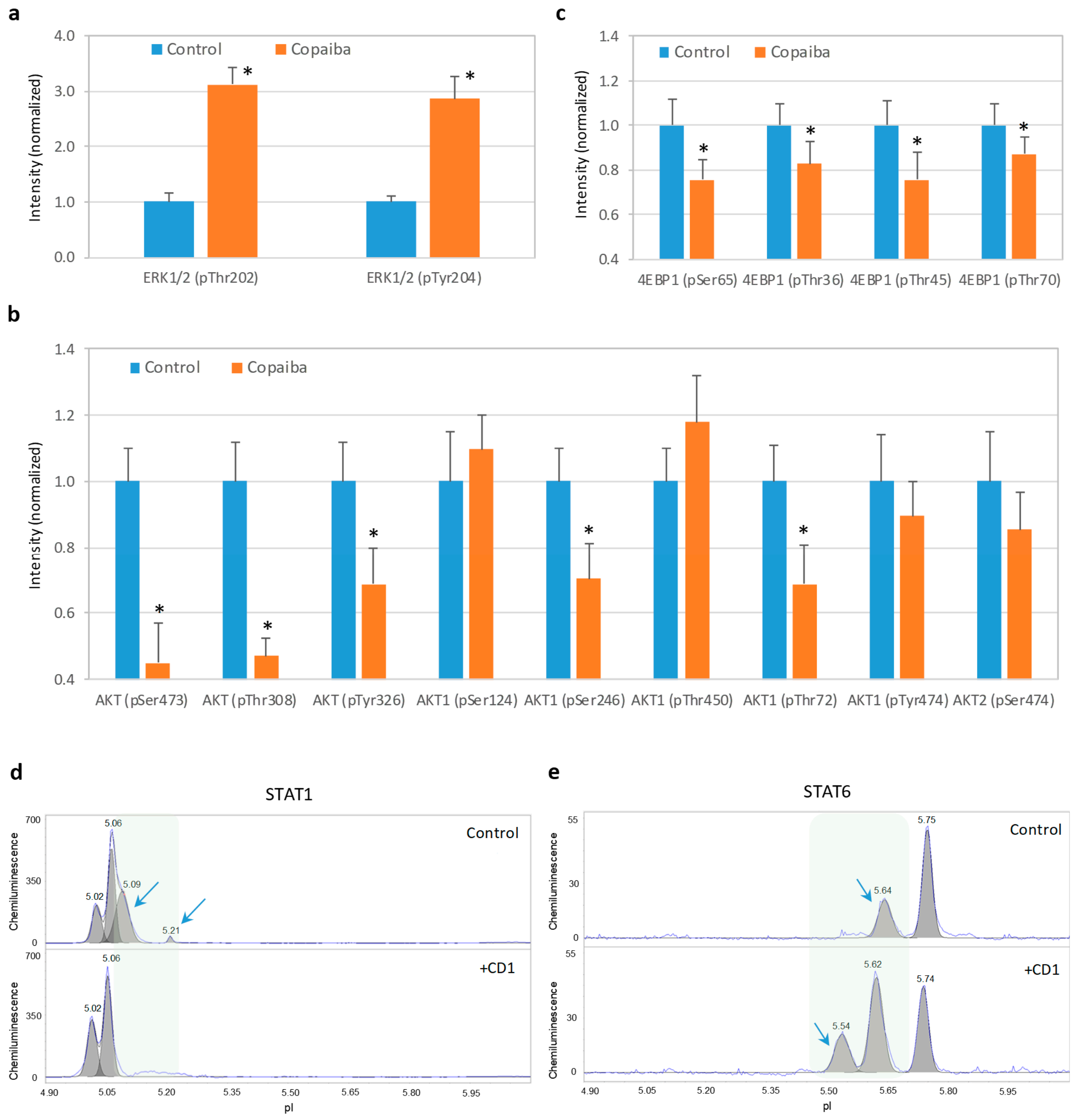

Supplemental Figure S2. Confirmation of nanofluidic protein PTM profiling data. (**a-c**) Phosphorylation profiling using antibody arrays. (**a**) Increased ERK1/2 (pThr202) and ERK1/2 (pTyr204) phosphor-isoforms following the treatment of HepG2 cells with copaiba essential oil. (**b**) Decreased Akt phosphor-isoforms including Akt (pSER473), Akt (pThr308), Akt (pTyr326), Akt1 (pSer326), and Akt1 (pThr72) following the treatment of HepG2 cells with copaiba essential oil. (**c**) Decreased 4EBP1 phosphor-isoforms including 4EBP1 (pSer65), 4EBP1 (pThr36), 4EBP1 (pThr45), 4EBP1 (pThr70) following the treatment of HepG2 cells with copaiba essential oil. Error bars are standard deviations across triplicate experiments. Asterisks indicate statistical significance with p-value <0.01 against untreated control. P-value was calculated using the paired Student's t-test. Antibody arrays were acquired from Full Moon Biosystems (Cat. No. PMT138, Sunnyvale, CA) and performed according to manufacturer's protocol. Array scanning and data analysis were performed by Full Moon Biosystem. (**d-e**) Detection of (**d**) increased STAT1 phosphorylation and (**e**) increased STAT6 phosphorylation with nanofluidic protein PTM profiling following the treatment of HepG2 cells with copaiba essential oil. Blue arrows and shaded backgrounds highlight areas where substantial changes to protein isoforms were observed.

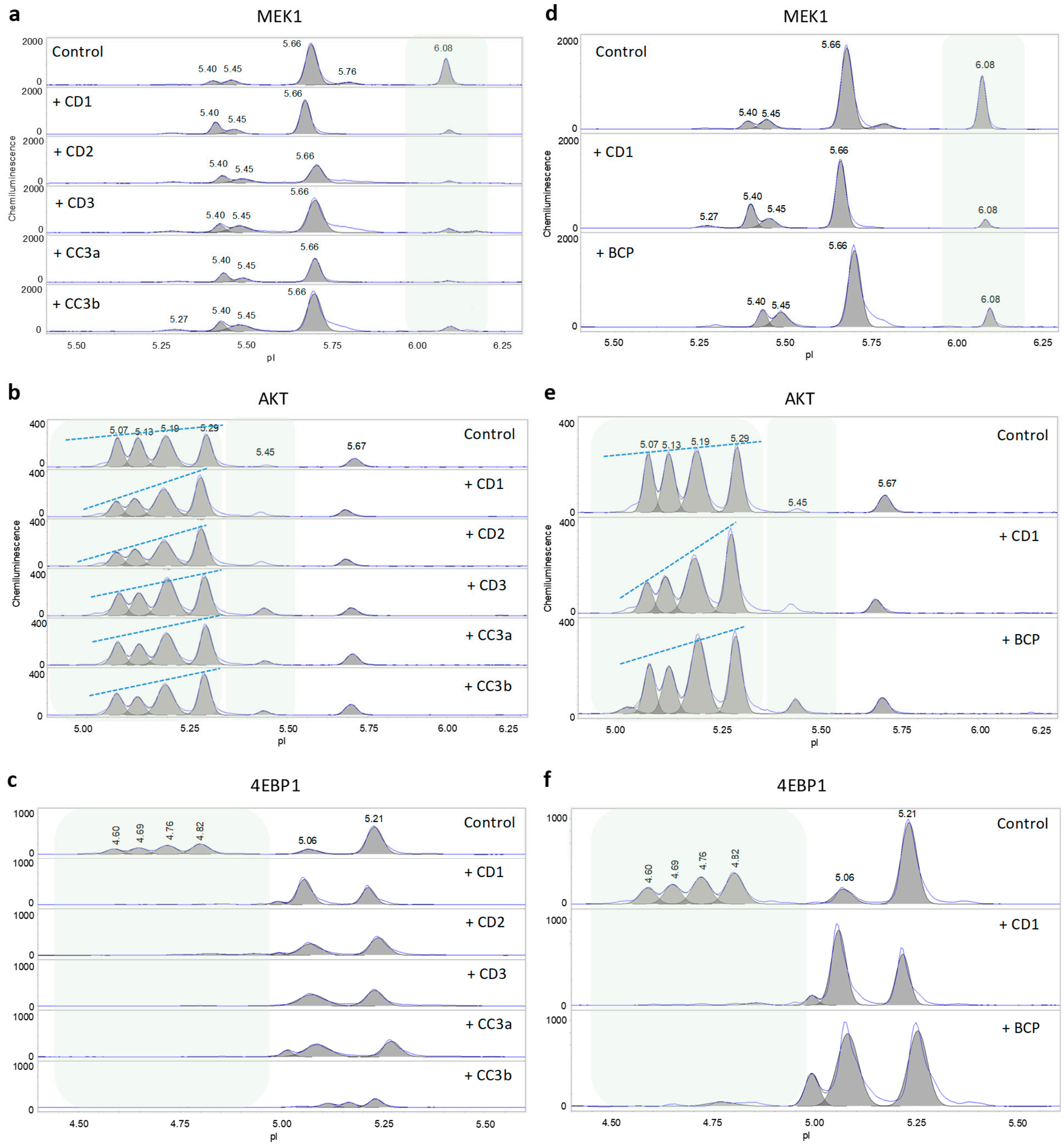

Supplemental Figure S3. Assessment of the effects of copaiba essential oil and its principle component on the signaling activities of HepG2 cells. (**a**-**c**) Consistency assessment of copaiba essential oils (CD1, CD2, CD3, CC3a, & CC3b) on the phosphorylation of MEK1 (**a**), Akt (**b**), and 4EBP1 (**c**). (**d**-**f**) β-caryophyllene could mimic the effects of copaiba essential oils on the phosphorylation of MEK1, Akt, and 4EBP1. Shaded backgrounds highlight areas where substantial changes to protein isoforms were observed. Dashed lines connect peak intensities to highlight changes to Akt phosphor-isoforms following the treatment of HepG2 cells with copaiba essential oil.

Supplemental Figures S4-S16. Chromatograms for essential oils.

Sample Information

Analyzed by	: Dr. Prabodh Satyal
Analyzed	: 10/9/2018 9:02:18 PM
Sample Type	: Essential Oil
Sample Name	: Green Mandarin (#1)
ARC#	: RU181008A SAP# 00000
Injection Volume	: 0.10
Data File	: C:\Users\user\Desktop\QA Data File\psatyal\Green Mandarin #1 APRC RU18100[illegible]

APRC
Aromatic Plant Research Center

Chromatogram Green Mandarin (#1) C:\Users\user\Desktop\QA Data File\psatyal\Green Mandarin #1 APRC RU181008A.qgd

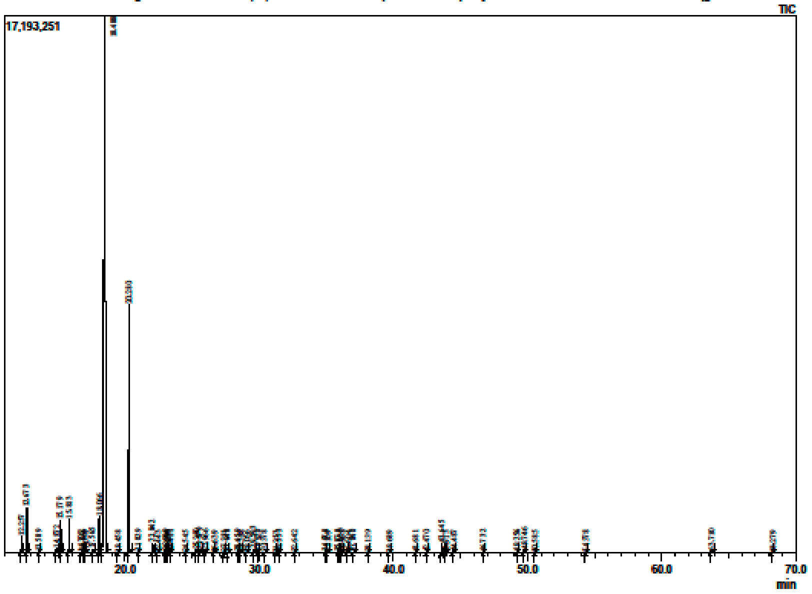

Comments:
PASS

Typical Ranges of Main Components:

Peak Report TIC

R.Time	Name	Area%
12.257	Thujene<alpha->	0.86
12.673	Pinene<alpha->	2.38
13.589	Camphene	0.02
14.872	Sabinene	0.29
15.179	Pinene<beta->	1.89
15.813	Myrcene	1.99
16.727	Octanal<n->	0.06
16.896	Phellandrene<alpha->	0.07
17.039	Carene<delta-3->	0.01
17.565	Terpinene<alpha->	0.19
18.066	Cymene<para->	2.97
18.488	Limonene	65.86
19.458	Ocimene<(E)-beta->	0.02
20.280	Terpinene <gamma>	19.43
21.029	Sabinene hydrate<cis->	0.04
22.042	Terpinolene	0.65
22.443	p-Cymenene+Epoxymyrcene	0.02
22.999	Linalool	0.15
23.096	Sabinene hydrate<trans->	0.08
23.205	Green Mandarin	0.01
23.411	Nonanal	0.01
24.545	Mentha-2,8-dien-1-ol<trans-para->	0.02
25.280	Limonene oxide<cis->	0.18
25.579	Limonene oxide<trans->	0.17
25.966	Terpinolene<epoxy>	0.15
26.639	Citronellal	0.01
27.381	2,6-Dimethyl-3,5,7-octatriene-2-ol, ,E,e-	0.07
27.648	Menthol	0.05
28.459	1,8-menthadien-4-ol <para>	0.02
28.588	Terpinen-4-ol	0.07
29.056	Cymen-8-ol<para->	0.03
29.593	Terpineol<alpha->	0.30
29.920	Green Mandarin	0.03
30.378	Decanal<n->	0.06
31.253	Carveol<trans->	0.01
31.553	Chrysanthenyl acetate<trans>	0.01
32.642	Ascaridole<Z>	0.02
34.978	Ascaridol glycol <E>	0.02
35.109	Perilla aldehyde	0.03
35.870	Anethole<E->	0.01
36.030	Thymol	0.11
36.162	Green Mandarin	0.04
36.604	Carvacrol	0.01
37.040	Ascaridole<E>	0.05
38.139	4-isopropyl-6-methyl-3-cyclohexene-1,2-diol	0.02
39.689	Menthene<1,2-diol>	0.04
41.681	Copaene<alpha->	0.02
42.470	Cubebene<beta->	0.01
43.645	Methyl anthranilate <methyl-n>	0.70
43.913	Dodecanal	0.02
44.487	Caryophyllene <trans->	0.11
46.732	Humulene <alpha>	0.01
49.256	Selinene <alpha>	0.07
49.746	Farnesene<(E,E)-alpha->	0.26
50.585	Cadinene <delta>	0.02
54.378	Caryophyllene oxide	0.03
63.710	Sinensal <alpha>	0.22
68.279	Neophytadiene	0.01
		100.00

Figure S4. Green mandarin essential oil CND

Sample Information

Analyzed by	: Dr. Prabodh Satyal
Analyzed	: 10/9/2018 10:58:11 PM
Sample Type	: Essential Oil
Sample Name	: Green Mandarin (#2)
ARC#	: RU181008B SAP# 00000
Injection Volume	: 0.10
Data File	: C:\Users\user\Desktop\QA Data File\psatyal\Green Mandarin #2 APRC RU18100[illegible]

Chromatogram Green Mandarin (#2) C:\Users\user\Desktop\QA Data File\psatyal\Green Mandarin #2 APRC RU181008B.qgd

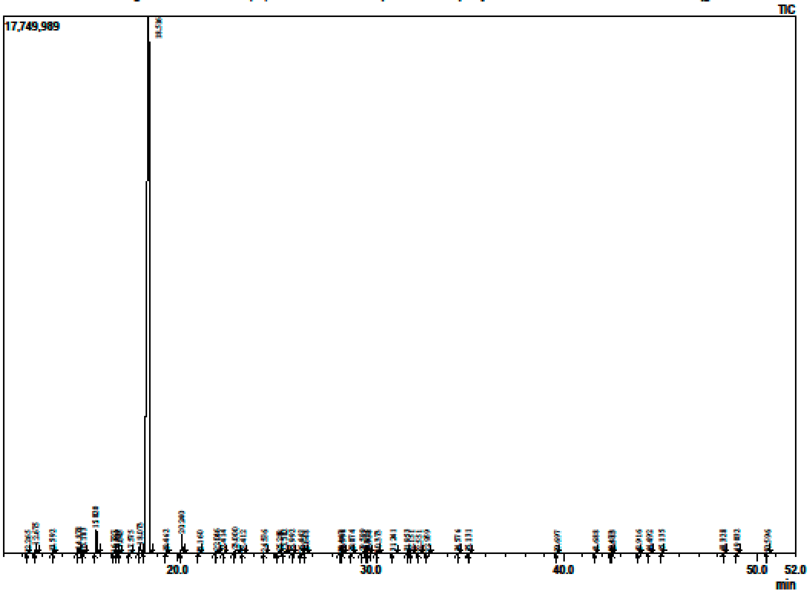

Peak Report TIC

R.Time	Name	Area%
12.265	Thujene<alpha->	0.02
12.675	Pinene<alpha->	0.60
13.592	Camphene	0.01
14.878	Sabinene	0.32
15.183	Pinene<beta->	0.12
15.820	Myrcene	1.75
16.727	Octanal<n->	0.10
16.899	Phellandrene<alpha->	0.05
17.043	Carene<delta-3->	0.10
17.575	Terpinene<alpha->	0.05
18.073	Cymene<para->	0.94
18.516	Limonene	92.56
19.462	Ocimene <E-beta>	0.03
20.240	Terpinene<gamma->	1.55
21.160	Pinol	0.02
22.046	Terpinolene	0.18
22.414	Cymenene <para->	0.02
23.000	Linalool	0.29
23.412	Nonanal	0.02
24.536	Mentha-2,8-dien-1-ol<trans-para->	0.06
25.289	Limonene oxide<cis->	0.13
25.582	Limonene oxide<trans->	0.14
25.992	Terpinolene<epoxy>	0.01
26.443	Terpineol<cis-beta->	0.02
26.648	Citronellal	0.03
28.467	1,8-menthadien-4-ol <para>	0.02
28.598	Terpinen-4-ol	0.03
29.074	Cymen-8-ol<para>	0.02
29.599	Terpineol<alpha->	0.18
29.825	Green Mandarin	0.01
29.938	Cis-Piperitol	0.05
30.373	Decanal	0.11
31.241	Carveol<trans->	0.07
31.953	Thymol, methyl ether	0.01
32.151	Carveol<cis->	0.04
32.551	Neral	0.02
32.959	Carvone	0.06
34.576	Geranial	0.03
35.131	Perilla aldehyde	0.01
39.697	Menthene<1,2-diol>	0.03
41.688	Copaene<alpha->	0.02
42.473	Cubebene <beta>	0.01
42.585	Elemene <beta>	0.01
43.916	Dodecanal	0.02
44.492	Caryophyllene <trans>	0.03
45.135	Copaene<beta->	0.01
48.328	Germacrene D	0.01
49.032	Valencene	0.05
50.596	Cadinene <delta->	0.03
		100.00

Comments:
PASS

Typical Ranges of Main Components:

Figure S5. Mandarin essential oil CNC1

Sample Information

Analyzed by	: Dr. Prabodh Satyal
Analyzed	: 10/10/2018 12:53:56 AM
Sample Type	: Essential Oil
Sample Name	: Tumeric (#1)
ARC#	: RU181008C SAP# 00000
Injection Volume	: 0.10
Data File	: C:\Users\user\Desktop\QA Data File\psatyal\Tumeric #1 APRC RU181008C.qgd

Chromatogram Tumeric (#1) C:\Users\user\Desktop\QA Data File\psatyal\Tumeric #1 APRC RU181008C.qgd

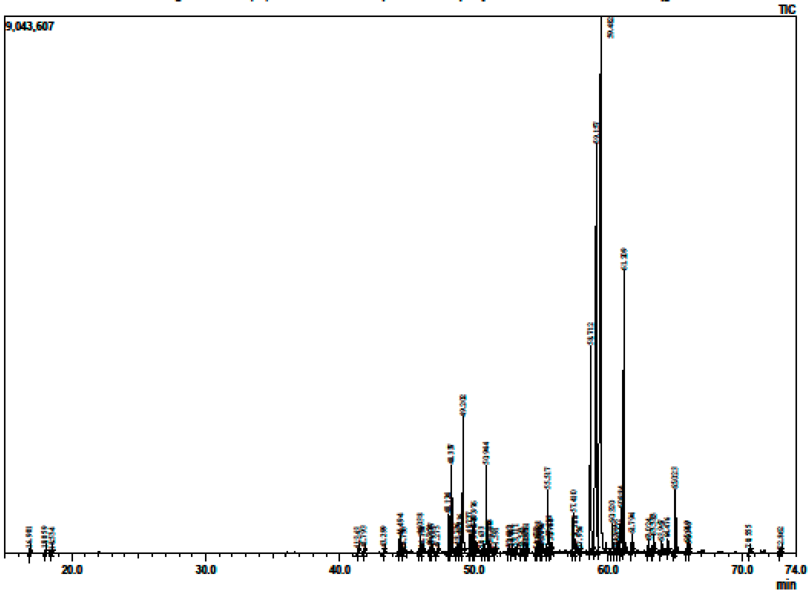

Comments:
PASS

Typical Ranges of Main Components:

Peak Report TIC

R.Time	Name	Area%
16.901	Phellandrene<alpha->	0.09
18.059	Cymene<para->	0.07
18.534	Cineole<1,8->	0.06
41.343	Megastigma-4,6(Z),8(Z)-triene	0.08
41.793	Turmeric	0.12
43.299	Turmeric	0.11
44.494	Caryophyllene <trans>	0.58
44.736	Turmeric	0.11
46.038	Turmeric	0.52
46.156	Turmeric	0.10
46.754	Humulene<alpha->	0.17
46.867	Amorpha-4,11-diene	0.22
47.275	Alloaromadendrene	0.13
48.124	Curcumene<gamma>	1.14
48.337	Curcumene <alpha>	2.66
48.736	Turmeric	0.24
49.016	Turmeric	0.50
49.202	Zingiberene<alpha>	4.42
49.727	Turmeric	0.55
49.976	Bisabolene <beta->	0.92
50.077	Curcumene <beta>	0.37
50.633	Turmeric	0.09
50.944	Sesquiphellandrene <beta>	2.75
51.110	Bisabolene <E-gamma>	0.28
51.188	Turmeric	0.32
51.591	Turmeric	0.09
52.612	Turmeric	0.11
52.802	Turmeric	0.14
53.111	Turmeric	0.14
53.543	Turmeric	0.10
53.856	Turmeric	0.11
53.951	Himachalene<beta-dehydro-Ar>	0.16
54.677	Turmeric	0.09
54.838	Cucumadione	0.29
54.956	Turmerone<ar-dihydro->	0.12
55.096	Turmeric	0.19
55.517	Helifolen-12-al B<anti-anti-anti>	2.28
55.636	Turmeric	0.43
55.766	Turmeric	0.28
57.410	Turmeric	1.35
57.588	Turmeric	0.47
57.956	Turmeric	0.08
58.712	Turmerone-alpha	7.37
59.157	Turmerone-Beta	23.56
59.482	Turmerone-Ar	27.31
60.320	Allantone<Z-gamma>	0.98
60.539	Turmeric	0.14
60.772	Turmeric	0.40
60.984	Allantone-<(E)-gamma->	2.05
61.209	Curlone	10.29
61.794	Allantone<Z-alpha>	0.69
63.024	Bisabolone <6R,7R>	0.34
63.383	Bisabolone <6S,7R->	0.53
63.967	Turmeric	0.33
64.476	Turmeric	0.35
65.023	Allantone <E-alpha>	1.95
65.919	Turmeric	0.25
66.046	Turmeric	0.19
70.555	Cyclohexanecarboxylic acid, 3-phenylpropyl ester	0.13
72.862	Turmeric	0.09
		100.00

Figure S6. Turmeric essential oil TD

Sample Information

Analyzed by	: Dr. Prabodh Satyal
Analyzed	: 10/10/2018 2:49:47 AM
Sample Type	: Essential Oil
Sample Name	: Tumeric (#2)
ARC#	: RU181008D SAP# 00000
Injection Volume	: 0.10
Data File	: C:\Users\user\Desktop\QA Data File\psatyal\Tumeric #2 APRC RU181008D.qgd

Chromatogram Tumeric (#2) C:\Users\user\Desktop\QA Data File\psatyal\Tumeric #2 APRC RU181008D.qgd

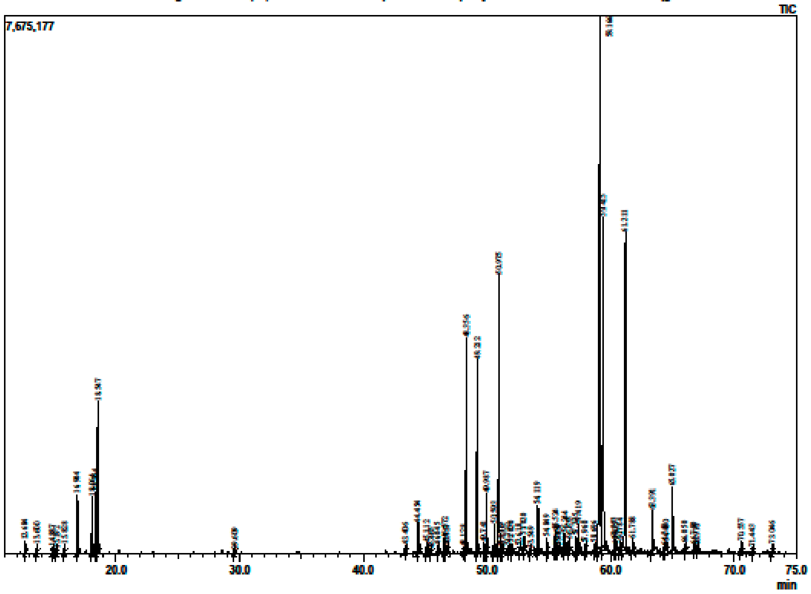

Comments:
PASS

Typical Ranges of Main Components:

Peak Report TIC

R.Time	Name	Area%
12.684	Pinene <alpha->	0.22
13.600	Camphene	0.18
14.887	Sabinene	0.13
15.192	Pinene <beta->	0.09
15.828	Myrcene	0.20
16.904	Phellandrene <alpha->	1.25
18.064	Cymene<para->	1.29
18.364	Limonene	1.44
18.547	1,8-cineole	3.76
29.609	Terpineol <alpha->	0.09
43.406	Sesquithujene	0.16
44.454	Santalene<alpha->	0.94
45.112	Elemene <gamma>	0.28
45.465	Turmeric	0.08
46.045	Turmeric	0.16
46.572	Farnesene<(E)-beta->	0.42
46.757	Humulene<alpha->	0.15
48.128	Curcumene<gamma>	0.12
48.356	Curcumene <alpha>	6.40
49.212	Zingiberene<alpha>	5.72
49.741	Turmeric	0.26
49.987	Bisabolene <beta->	1.71
50.593	Teresantalic acid <alpha>	0.82
50.975	Sesquiphellandrene <beta>	8.67
51.123	Bisabolene <E-gamma>	0.21
51.210	Turmeric	0.15
51.732	Guaia-3,9-diene	0.17
52.020	Selina-3,7(11)-diene	0.24
52.618	Turmeric	0.09
53.020	Germacrene B	0.39
53.549	1,2,3,1',2',3'-Hexamethyl-bicyclopentyl-2,2'-diene	0.10
54.119	Turmerol<ar->	1.36
54.849	Turmeric	0.39
55.524	Helifolen-12-al B<anti-anti-anti>	0.66
55.660	Turmeric	0.14
55.857	Atlantol<beta->	0.24
56.244	Zingiberenol	0.52
56.633	Helifolen-12-al C<anti-syn-syn>	0.35
57.225	Sesquisabinene hydrate	0.46
57.419	Turmeric	0.86
57.960	Turmeric	0.18
58.696	Turmerone-alpha	0.26
59.166	Turmerone-Beta	27.75
59.415	Turmerone-Ar	13.30
60.333	Himachal-4-en-1-beta-ol <11-beta-H>	0.39
60.435	Turmeric	0.15
60.784	Farnesol <(2Z,6Z)->	0.37
61.211	Curlone	11.89
61.788	Atlantone<(Z)-alpha->	0.42
63.391	Bisabolone <6S,7R>	1.19
64.351	Turmeric	0.14
64.480	Turmeric	0.24
65.027	Atlantone <E-alpha>	1.81
66.050	Turmeric	0.17
66.780	Epoxy Zingiberene	0.13
66.976	Turmeric	0.10
70.557	Cyclohexanecarboxylic acid, 3-phenylpropyl ester	0.32
71.443	(S)-3-Methyl-6-((S)-6-methyl-4-oxohept-5-en-2-yl)cy	0.12
73.046	Turmeronol A	0.22
		100.00

Figure S7. Turmeric essential oil TC2

Sample Information

Analyzed by	: Dr. Prabodh Satyal
Analyzed	: 10/10/2018 6:41:20 AM
Sample Type	: Essential Oil
Sample Name	: Melissa (#1)
ARC#	: RU181008E SAP# 00000
Injection Volume	: 0.10
Data File	: C:\Users\user\Desktop\QA Data File\psatyal\Melissa #1 APRC RU181008E.qgd

APRC
Aromatic Plant Research Center

Chromatogram Melissa (#1) C:\Users\user\Desktop\QA Data File\psatyal\Melissa #1 APRC RU181008E.qgd

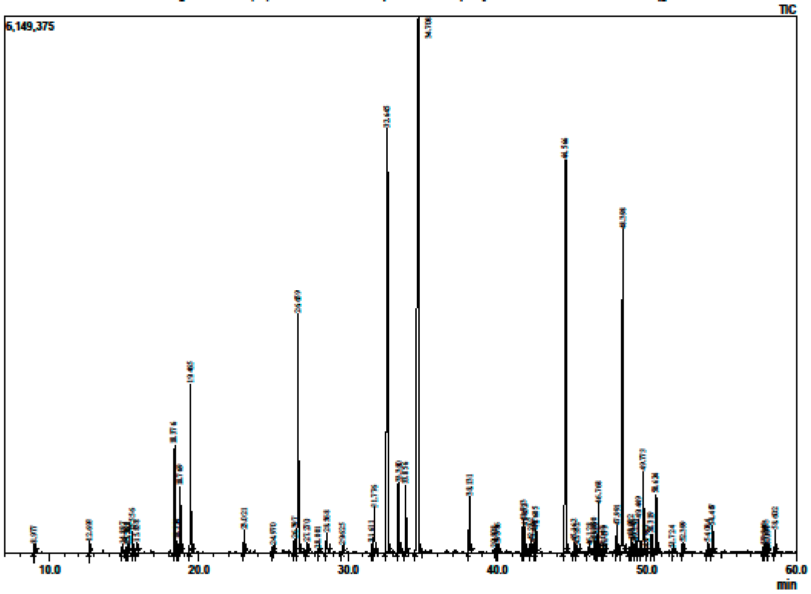

Comments:
PASS

Typical Ranges of Main Components:

Peak Report TIC

R.Time	Name	Area%
8.977	Hexenol <3Z>	0.20
12.693	Pinene <alpha>	0.21
14.897	Sabinene	0.17
15.202	Pinene <beta->	0.15
15.268	Octen-3-ol<1->	0.15
15.556	Hept-5-en-2-one <6-methyl->	0.46
15.838	Myrcene	0.26
18.376	Limonene	2.30
18.554	1,8-cineole	0.26
18.769	Ocimene <Z-beta>	1.41
19.465	Ocimene <E-beta>	3.67
23.021	Linalool	0.51
24.970	Ocimene<allo->	0.17
26.397	Chrysanthemal <trans>	0.34
26.659	Citronellal	5.86
27.270	Isocitral <cis>	0.25
28.001	Mentha-1,5-dien-8-ol<para->	0.08
28.568	Isocitral <E->	0.61
29.625	Terpineol <alpha>	0.20
31.611	Nerol	0.23
31.776	Citronellol	1.23
32.645	Neral	15.30
33.380	Geraniol	1.82
33.856	Citronellate <methyl->	1.68
34.708	Geranial	23.53
38.131	Geranate <methyl->	1.41
39.808	Cubebene<alpha->	0.05
39.986	Citronellyl acetate	0.19
41.713	Copaene <alpha>	0.81
41.852	Geranyl acetate	0.68
42.232	Bourbonene <alpha>	0.30
42.496	Cubebene <beta>	0.23
42.605	Elemene <beta>	0.60
44.566	Caryophyllene <trans>	12.30
45.162	Copaene <beta>	0.28
45.352	Bergamotene <trans-alpha>	0.20
46.128	Cinnamyl acetate <trans>	0.23
46.490	Muurola-3,5-diene <trans>	0.28
46.589	Farnesene<(E)-beta->	0.15
46.768	Humulene <alpha>	1.35
47.047	Alloaromadendrene	0.10
47.179	Cadina-1(6),4-diene <cis>	0.12
47.991	Cadina-1(6),4-diene <trans->	0.73
48.398	Germacrene D	9.88
48.932	Isobicyclogermacrene	0.45
49.043	Muurola-4(14),5-diene <trans>	0.32
49.250	Bicyclogermacrene	0.32
49.449	Muurolene<alpha->	1.03
49.773	Farnesene <(E,E)alpha>	2.25
49.995	Bisabolene <beta->	0.09
50.319	Amorphene <delta>	0.44
50.624	Cadinene <delta>	1.54
51.724	Cadinene <alpha>	0.10
52.399	Elemol <alpha->	0.24
54.084	Germacrene D-4-ol	0.29
54.407	Caryophyllene oxide	0.77
57.846	Muurolol <alpha>	0.22
57.953	Muurolol <epi-alpha>	0.28
58.097	Cadinol <delta>	0.10
58.602	Cadinol <alpha>	0.64
		100.00

Figure S8. Melissa essential oil MD1

Sample Information

Analyzed by	: Dr. Prabodh Satyal
Analyzed	: 10/10/2018 8:37:01 AM
Sample Type	: Essential Oil
Sample Name	: Melissa (#2)
ARC#	: RU181008F SAP# 00000
Injection Volume	: 0.10
Data File	: C:\Users\user\Desktop\QA Data File\psatyal\Melissa #2 APRC RU181008F.qgd

APRC
Aromatic Plant Research Center

Chromatogram Melissa (#2) C:\Users\user\Desktop\QA Data File\psatyal\Melissa #2 APRC RU181008F.qgd

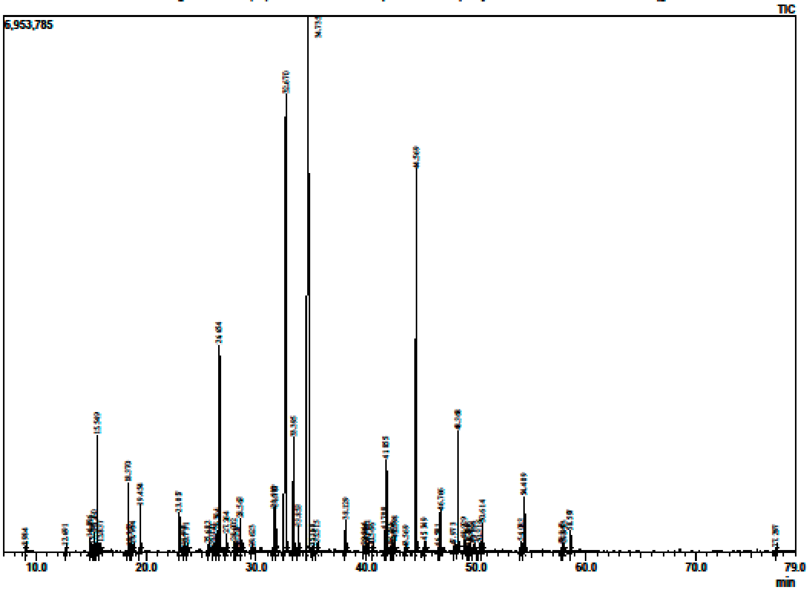

Comments:
PASS

Typical Ranges of Main Components:

Peak Report TIC

R.Time	Name	Area%
8.984	Hexenol <3Z>	0.13
12.691	Pinene <alpha>	0.09
14.896	Sabinene	0.29
15.201	Pinene <beta->	0.11
15.260	Octen-3-ol<1->	0.43
15.549	Hept-5-en-2-one <6-methyl->	2.64
15.837	Myrcene	0.20
18.370	Limonene	1.54
18.552	1,8-cineole	0.12
18.764	Ocimene <Z-beta>	0.19
19.454	Ocimene <E-beta>	1.06
23.017	Linalool	0.95
23.401	Nonanal	0.18
23.731	Rose oxide <cis>	0.12
25.682	Epiphotocitral A	0.16
26.020	Isocitral <exo>	0.12
26.394	Chrysanthemal <trans>	0.55
26.654	Citronellal	5.44
27.264	Isocitral <cis>	0.46
28.002	Borneol	0.27
28.229	Pinocamphone+Necrodol	0.08
28.563	Isocitral <E->	1.06
29.623	Terpineol <alpha>	0.09
31.610	Nerol	1.24
31.787	Citronellol	1.37
32.670	Neral	19.92
33.395	Geraniol	3.37
33.853	Citronellate <methyl->	0.67
34.735	Geranial	28.43
35.168	Nerate<methyl->	0.09
35.515	Carvone oxide<E>	0.20
38.129	Geranate <methyl->	0.86
39.844	Neric acid	0.13
39.984	Citronellyl acetate	0.09
40.121	Eugenol	0.21
40.566	Neryl acetate	0.12
41.708	Copaene <alpha>	0.57
41.855	Geranyl acetate	2.53
42.231	Bourbonene <alpha>	0.15
42.495	Cubebene <beta>	0.18
42.598	Elemene <beta>	0.39
43.569	Caryophyllene <cis>	0.09
44.569	Caryophyllene <trans>	12.89
45.349	Bergamotene <trans-alpha>	0.29
46.581	Farnesene<(E)-beta->	0.10
46.766	Humulene <alpha->	1.20
47.973	Cadina-1(6),4-diene <trans>	0.14
48.368	Germacrene D	3.61
48.929	Isobicyclogeremacrene	0.27
49.246	Bicyclogermacrene	0.12
49.432	Muurolene <alpha>	0.21
49.766	Farnesene <(E,E)alpha>	0.19
50.316	Amorphene <delta>	0.20
50.614	Cadinene <delta->	0.80
54.083	Germacrene D-4-ol	0.30
54.409	Caryophyllene oxide	1.69
57.845	Muurolol <alpha>	0.19
57.953	Muurolol <epi-alpha>	0.24
58.597	Cadinol <alpha>	0.57
77.297	Pseudo phytol<6Z,10Z->	0.11
		100.00

Figure S9. Melissa essential oil MD2

Sample Information

**Analyzed by**	: Dr. Prabodh Satyal
**Analyzed**	: 10/10/2018 10:32:56 AM
**Sample Type**	: Essential Oil
**Sample Name**	: Melissa (#3)
**ARC#**	: RU181008G SAP# 00000
**Injection Volume**	: 0.10
**Data File**	: C:\Users\user\Desktop\QA Data File\psatyal\Melissa #3 APRC RU181008G.qgd

APRC
Aromatic Plant Research Center

Chromatogram Melissa (#3) C:\Users\user\Desktop\QA Data File\psatyal\Melissa #3 APRC RU181008G.qgd

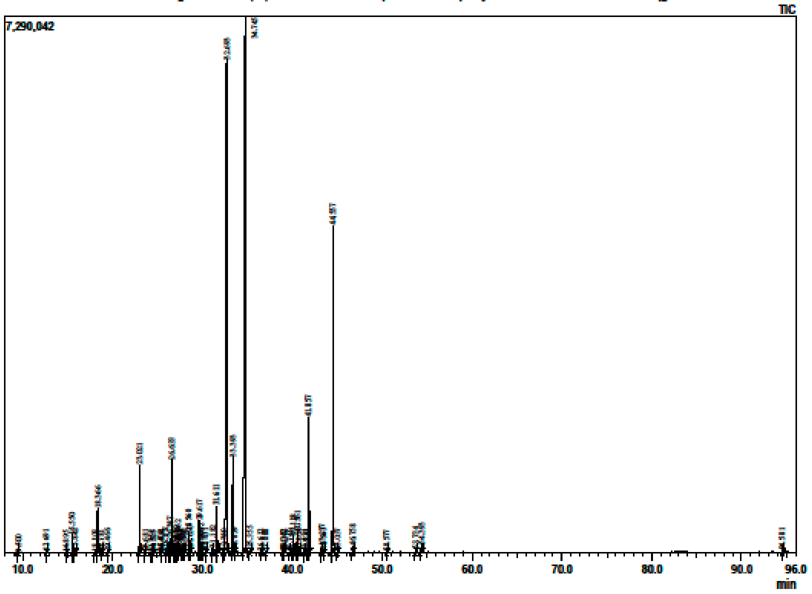

Comments:
PASS

Typical Ranges of Main Components:

Peak Report TIC

R.Time	Name	Area%
9.500	1-Isopropyl-3-methylcyclopentane	0.01
12.691	Pinene-<alpha->	0.01
14.895	Sabinene	0.00
15.550	Hept-5-en-2-one <6-methyl->	0.49
15.842	Myrcene	0.10
18.103	Cymene <para->	0.06
18.366	Limonene	1.21
18.781	Ocimene <Z-beta>	0.01
19.466	Ocimene-<(E)-beta->	0.06
23.021	Linalool	2.52
23.681	Cyclocitral alpha	0.04
24.358	Fenchol <endo>	0.02
24.525	Chrysanthenone	0.02
25.402	Linalool <dihydro>	0.02
25.514	Terpin-3-en-1-ol	0.03
26.017	Isocitral <exo>	0.13
26.387	Isopulegol-iso->	0.48
26.633	Citronellal	2.82
26.918	Menthone	0.02
27.080	Isopulegol	0.14
27.262	Isocitral <cis>	0.34
27.415	Lavandulol	0.03
27.713	Terpineol <trans-beta>	0.01
27.825	Isopulegol-neoiso->	0.05
28.001	Borneol	0.03
28.560	Isocitral <E->	0.68
28.650	Anethofuran+Piperitol-Z>	0.13
29.617	Terpineol <alpha>	1.00
29.809	Melissa	0.11
29.952	Terpineol<gamma->	0.24
30.431	Cyclocitral beta	0.07
31.182	Isopiperitenol-trans->	0.19
31.611	Nerol	1.63
32.380	Butenyl tiglate 3-methyl-3	0.06
32.693	Neral	28.97
33.393	Geraniol	3.42
33.659	Piperitone	0.07
34.745	Geranial	35.33
35.355	Melissa	0.16
36.612	Geranyl formate	0.02
37.088	Citronellic acid	0.02
39.042	Melissa	0.03
39.122	Melissa	0.03
39.740	Neric acid	0.04
40.118	Eugenol	0.53
40.561	Neryl acetate	0.64
40.808	5E)-2,4,4,7-tetramethylocta-5,7-dien-3-ol	0.04
41.421	Melissa	0.04
41.703	Copaene <alpha>	0.02
41.857	Geranyl acetate	4.41
43.277	Methyleugenol	0.22
43.563	Caryophyllene <cis>	0.05
44.557	Caryophyllene <trans>	12.44
45.037	Ylangene <beta>	0.05
46.758	Humulene <alpha>	0.21
50.577	Melissa	0.02
53.734	Melissa	0.11
54.393	Caryophyllene oxide	0.35
94.581	Monolinolein	0.03
		100.00

Figure S10. Melissa essential oil MC3a

Sample Information

Analyzed by	: Dr. Prabodh Satyal
Analyzed	: 10/10/2018 12:28:43 PM
Sample Type	: Essential Oil
Sample Name	: Melissa (#4)
ARC#	: RU181008H SAP# 00000
Injection Volume	: 0.10
Data File	: C:\Users\user\Desktop\QA Data File\psatyal\Melissa #4 APRC RU181008H.qgd

APRC Aromatic Plant Research Center

Chromatogram Melissa (#4) C:\Users\user\Desktop\QA Data File\psatyal\Melissa #4 APRC RU181008H.qgd

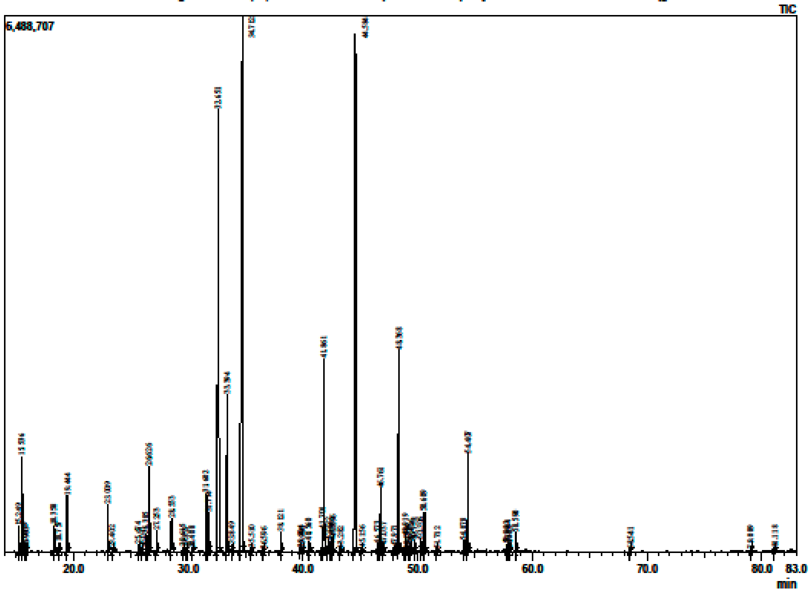

Comments:
PASS

Typical Ranges of Main Components:

Peak Report TIC

R.Time	Name	Area%
15.249	1-Octen-3-ol	0.50
15.536	Hept-5-en-2-one <6-methyl->	2.02
15.829	Myrcene	0.15
15.905	Cineole<dehydro-1,8->	0.05
18.358	Limonene	0.53
18.757	Ocimene <Z-beta>	0.18
19.444	Ocimene <E-beta>	1.23
23.009	Linalool	1.08
23.402	Nonanal<n->	0.13
25.674	Epiphotocitral A	0.18
26.011	Isocitral <exo>	0.10
26.385	Chrysanthenal<E>	0.41
26.626	Citronellal	2.08
27.253	Isocitral <cis>	0.51
28.553	Isocitral <E->	0.93
29.615	Terpineol <alpha>	0.09
29.827	Methyl chavicol	0.08
30.408	Cyclocitral beta	0.08
31.602	Nerol	1.53
31.774	Citronellol	1.10
32.651	Neral	17.26
33.394	Geraniol	4.50
33.849	Citronellate <methyl->	0.17
34.712	Geranial	24.54
35.510	Carvone oxide<E>	0.11
36.596	Geranyl formate	0.07
38.121	Geranate <methyl->	0.50
39.804	Neric acid	0.07
39.980	Citronellyl acetate	0.07
40.560	Neryl acetate	0.26
41.701	Copaene <alpha>	0.60
41.861	Geranyl acetate	5.20
42.222	Bourbonene <alpha>	0.29
42.486	Cubebene <beta>	0.19
42.596	Elemene <beta>	0.40
43.282	Methyleugenol	0.10
44.584	Caryophyllene <trans>	17.82
45.156	Copaene <beta>	0.11
46.573	Farnesene<(E)-beta->	0.18
46.761	Humulene <alpha>	1.74
47.037	Alloaromadendrene	0.19
47.971	Cadina-1(6),4-diene <trans->	0.13
48.368	Germacrene D	5.67
48.919	Isobicyclogeremacrene	0.42
49.030	Muurola-4(14),5-diene <trans>	0.06
49.239	Bicyclogermacrene	0.14
49.426	Muurolene <alpha>	0.26
49.758	Farnesene <(E,E)alpha>	0.28
50.306	Amorphene <delta>	0.30
50.609	Cadinene <delta->	1.02
51.712	Cadinene <alpha>	0.07
54.078	Germacrene D-4-ol	0.29
54.407	Caryophyllene oxide	2.86
57.833	Muurolol <alpha>	0.18
57.944	Muurolol <epi-alpha>	0.24
58.085	Cadinol <delta>	0.07
58.590	Cadinol <alpha>	0.51
68.541	Phytone	0.06
79.089	Ricenelidic acid lactone	0.07
81.118	6-Methyl-4,6-bis(4-methylpent-3-en-1-yl)cyclohexa-	0.08
		100.00

Figure S11. Melissa essential oil MC3b

Sample Information

Analyzed by : Dr. Prabodh Satyal
Analyzed : 10/9/2018 5:18:52 PM
Sample Type : Essential Oil
Sample Name : Copaiba (#1)
ARC# : RU181008J SAP# 00000
Injection Volume : 0.10
Data File : C:\Users\user\Desktop\QA Data File\psatyal\Copaiba #1 APRC RU181008J.qgd

APRC
Aromatic Plant Research Center

Chromatogram Copaiba (#1) C:\Users\user\Desktop\QA Data File\psatyal\Copaiba #1 APRC RU181008J.qgd

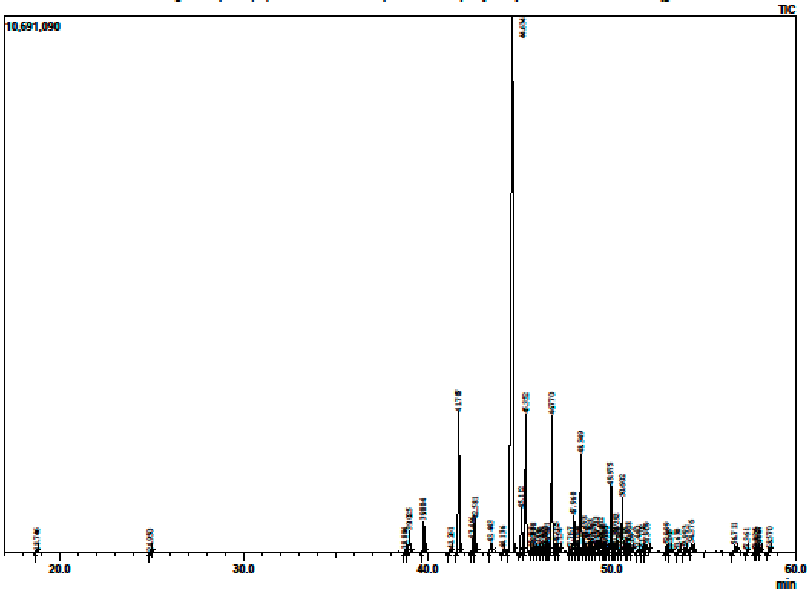

Comments:
PASS

Typical Ranges of Main Components:

Peak Report TIC

R.Time	Name	Area%
18.746	Ocimene-<(Z)-beta->	0.10
24.950	Ocimene <allo>	0.04
38.804	Bicycloelemene	0.13
39.025	Elemene <delta->	1.12
39.804	Cubebene <alpha>	1.51
41.261	Ylangene-<alpha->	0.21
41.707	Copaene <alpha>	7.19
42.466	Cubebene <beta>	0.68
42.581	Elemene <beta>	1.67
43.443	Cyperene	0.46
44.136	Bergamotene-<alpha-cis->	0.13
44.634	Caryophyllene <trans>	45.24
45.112	Elemene <gamma>	2.26
45.352	Bergamotene <trans-alpha>	7.19
45.688	Aromadendrene	0.16
45.808	Farnesene <Z-beta->	0.31
46.006	Humulene-<beta->	0.11
46.219	Santalene-<epi-beta->	0.13
46.352	Murrola-3,5-diene <cis->	0.08
46.473	Murrola-3,5-diene <trans>	0.16
46.561	Farnesene-<(E)-beta->	0.32
46.770	Humulene <alpha->	7.18
47.025	Alloaromadendrene	0.45
47.154	Muurola-4(14),5-diene <cis>	0.09
47.767	Cadina-1(6),4-diene-<cis->	0.13
47.960	Cadina-1(6),4-diene <trans->	2.03
48.112	Curcumene-<gamma>	0.14
48.218	Amorphene <alpha>	0.18
48.349	Germacrene D	5.16
48.498	Farnesene <alpha-cis-cis->	0.72
48.694	Selinene <delta>	0.09
48.828	Selinene <beta->	0.55
49.027	Muurola-4(14),5-diene <trans>	0.47
49.233	Bicyclogermacrene	0.93
49.412	Muurolene <alpha>	0.76
49.521	Bisabolene <cis-alpha->	0.32
49.680	Cadinene <beta->	0.13
49.735	Copaiba	0.16
49.975	Bisabolene <beta->	3.47
50.150	Bisabolene <Z-gamma>	0.24
50.292	Amorphene <delta>	0.83
50.444	Copaiba	0.10
50.602	Cadinene <delta->	2.78
50.795	Calamenene <trans>	0.10
50.918	Sesquiphellandrene <beta>	0.56
51.092	Bisabolene <E-gamma>	0.06
51.440	Cadina-1,4-diene <trans->	0.14
51.702	Cadinene-<alpha->	0.26
51.909	Bisabolene <trans-alpha>	0.52
52.999	Germacrene B	0.36
53.205	Calacorene-<beta>	0.07
53.630	Maaliol	0.07
53.993	Caryophyllene alcohol	0.27
54.376	Caryophyllene oxide	0.52
56.711	Humulane-1,6-dien-3-ol	0.42
57.361	Copaiba	0.05
57.825	Muurolol <alpha>	0.06
57.922	Muurolol <epi-alpha>	0.12
58.067	Cadinol <delta>	0.16
58.570	Cadinol-<alpha->	0.16
		100.00

Figure S12. Copaiba essential oil CD1

Sample Information

Analyzed by	: Dr. Prabodh Satyal
Analyzed	: 10/9/2018 3:23:07 PM
Sample Type	: Essential Oil
Sample Name	: Copaiba (#2)
ARC#	: RU181008K SAP# 00000
Injection Volume	: 0.10
Data File	: C:\Users\user\Desktop\QA Data File\psatyal\Copaiba #2 APRC RU181008K.qgd

Chromatogram Copaiba (#2) C:\Users\user\Desktop\QA Data File\psatyal\Copaiba #2 APRC RU181008K.qgd

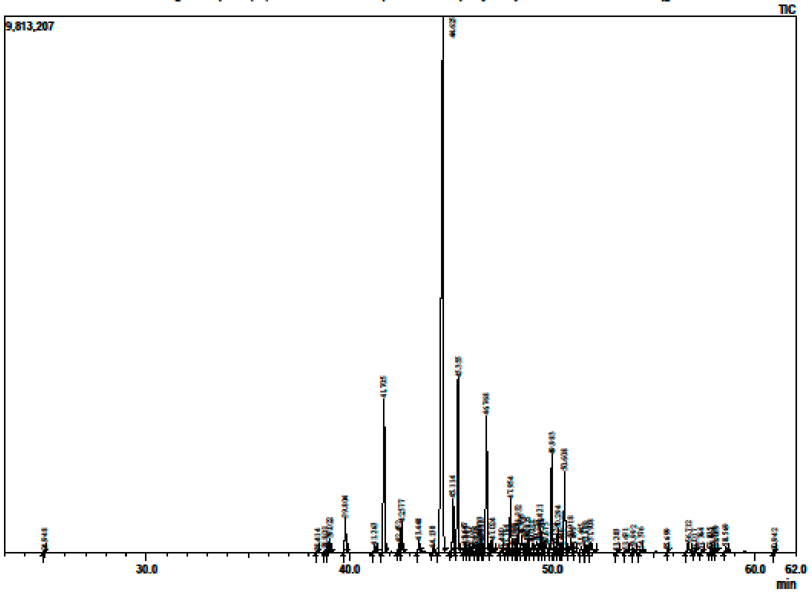

Comments:
PASS

Typical Ranges of Main Components:

Peak Report TIC

R.Time	Name	Area%
24.948	Ocimene <allo>	0.06
38.414	Bicyclo<nonene>	0.06
38.803	Bicycloelemene	0.14
39.022	Elemene <delta->	0.70
39.804	Cubebene <alpha>	1.56
41.263	Ylangene<alpha->	0.37
41.705	Copaene <alpha>	7.43
42.452	Sesquithujene<7-epi->	0.45
42.577	Elemene <beta>	1.44
43.440	Cyperene	0.51
44.130	Bergamotene<alpha-cis->	0.16
44.625	Caryophyllene <trans>	41.89
45.114	Elemene<gamma->	2.82
45.355	Bergamotene <trans-alpha>	8.51
45.687	Aromadendrene	0.39
45.806	Farnesene-Z-beta	0.12
46.035	Humulene<beta>	0.16
46.216	Santalene <beta-, epi->	0.15
46.366	Muurola-3,5-diene<cis->	0.13
46.473	Murrola-3,5-diene <trans>	0.66
46.552	Farnesene<(E)-beta->	0.40
46.768	Humulene <alpha->	6.67
47.024	Alloaromadendrene	0.64
47.480	Elemene <isomer>	0.05
47.764	Cadina-1(6),4-diene <cis->	0.23
47.954	Cadina-1(6),4-diene <trans->	2.67
48.111	Curcumene<gamma>	0.23
48.208	Amorphene <alpha>	0.25
48.332	Germacrene D	1.20
48.495	Farnesene <alpha-cis-cis->	0.84
48.695	Selinene <delta>	0.08
48.825	Selinene <beta->	0.63
49.033	Amorphene <gamma>	0.65
49.257	Selinene <alpha>	0.80
49.421	Muurolene <alpha>	1.35
49.516	Bisabolene <cis-alpha->	0.46
49.675	Cadinene <beta->	0.48
49.983	Bisabolene <beta->	4.89
50.152	Bisabolene<(Z)-gamma->	0.26
50.294	Amorphene <delta>	1.18
50.446	Copaiba	0.09
50.608	Cadinene <delta->	3.88
50.918	Sesquiphellandrene <beta>	0.84
51.096	Bisabolene <E-gamma>	0.07
51.445	Cadina-1,4-diene <trans->	0.29
51.699	Cadinene <alpha>	0.35
51.908	Bisabolene <trans-alpha>	0.71
53.203	Calcorene<beta>	0.05
53.631	Maaliol	0.09
53.992	Caryophyllene alcohol	0.23
54.376	Caryophyllene oxide	0.17
55.699	Ledol	0.04
56.712	Humulane-1,6-dien-3-ol	0.58
57.017	Cubenol<1-epi>	0.06
57.364	Copaiba	0.07
57.825	Muurolol <alpha>	0.09
57.924	Muurolol <epi-alpha>	0.16
58.069	Cadinol <delta>	0.23
58.569	Cadinol<alpha->	0.29
60.942	Juniper camphor	0.06
		100.00

Figure S13. Copaiba essential oil CD2

Sample Information

Analyzed by	: Dr. Prabodh Satyal
Analyzed	: 10/9/2018 1:27:09 PM
Sample Type	: Essential Oil
Sample Name	: Copaiba (#3)
ARC#	: RU181008L SAP# 00000
Injection Volume	: 0.10
Data File	: C:\Users\user\Desktop\QA Data File\psatyal\Copaiba #3 APRC RU181008L.qgd

APRC
Aromatic Plant Research Center

Chromatogram Copaiba (#3) C:\Users\user\Desktop\QA Data File\psatyal\Copaiba #3 APRC RU181008L.qgd

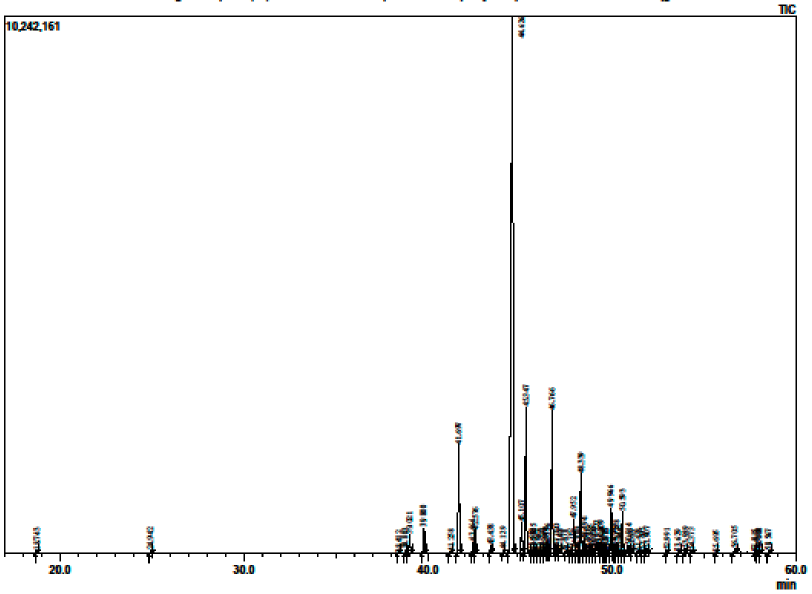

Comments:
PASS

Typical Ranges of Main Components:

Peak Report TIC

R.Time	Name	Area%
18.743	Ocimene<(Z)-beta->	0.15
24.942	Ocimene <allo>	0.15
38.412	Bicyclo<nonene>	0.03
38.803	Bicycloelemene	0.15
39.021	Elemene <delta->	1.06
39.800	Cubebene <alpha>	1.33
41.258	Ylangene<alpha->	0.14
41.697	Copaene <alpha>	5.97
42.464	Cubebene <beta>	0.61
42.576	Elemene <beta>	1.28
43.438	Cyperene	0.38
44.129	Bergamotene<alpha-cis->	0.14
44.626	Caryophyllene <trans>	49.10
45.107	Elemene <gamma>	1.76
45.347	Bergamotene <trans-alpha>	8.30
45.683	Aromadendrene	0.11
45.805	Farnesene-Z-beta	0.36
45.987	Humulene<beta->	0.13
46.212	Santalene <beta-, epi->	0.14
46.349	Muurola-3,5-diene<cis->	0.08
46.470	Murrola-3,5-diene <trans>	0.15
46.556	Farnesene<(E)-beta->	0.34
46.766	Humulene <alpha>	8.25
47.020	Alloaromadendrene	0.39
47.148	Muurola-4(14),5-diene <cis>	0.07
47.478	Elemene <isomer>	0.06
47.762	Cadina-1(6),4-diene<cis->	0.15
47.952	Cadina-1(6),4-diene <trans>	1.98
48.111	Curcumene<gamma>	0.15
48.213	Amorphene <alpha>	0.13
48.339	Germacrene D	4.57
48.494	Farnesene <alpha-cis-cis->	0.82
48.691	Selinene <delta>	0.08
48.825	Selinene <beta->	0.39
49.020	Muurola-4(14),5-diene <trans>	0.39
49.222	Bicyclogermacrene	0.83
49.409	Muurolene <alpha>	0.68
49.513	Bisabolene <cis-alpha->	0.20
49.673	Cadinene <beta->	0.14
49.735	Copaiba	0.10
49.966	Bisabolene <beta->	2.52
50.145	Bisabolene <Z-gamma>	0.37
50.288	Amorphene <delta>	0.62
50.444	Copaiba	0.09
50.593	Cadinene <delta>	2.30
50.914	Sesquiphellandrene <beta>	0.65
51.089	Bisabolene <E-gamma>	0.08
51.438	Cadine-1,4-diene <trans->	0.12
51.695	Cadinene<alpha->	0.18
51.907	Bisabolene <trans-alpha>	0.37
52.991	Germacrene B	0.16
53.629	Maaliol	0.06
53.989	Caryophyllene alcohol	0.31
54.373	Caryophyllene oxide	0.18
55.695	Ledol	0.04
56.705	Humulane-1,6-dien-3-ol	0.32
57.815	Muurolol <alpha>	0.05
57.919	Muurolol <epi-alpha>	0.08
58.068	Cadinol <delta>	0.14
58.567	Cadinol<alpha->	0.11
		100.00

Figure S14. Copaiba essential oil CD3

Sample Information

**Analyzed by**	: Dr. Prabodh Satyal
**Analyzed**	: 10/9/2018 11:31:21 AM
**Sample Type**	: Essential Oil
**Sample Name**	: Copaiba (#4)
**ARC#**	: RU181008M SAP# 00000
**Injection Volume**	: 0.10
**Data File**	: C:\Users\user\Desktop\QA Data File\psatyal\Copaiba #4 APRC RU181008M.qgd

Chromatogram Copaiba (#4) C:\Users\user\Desktop\QA Data File\psatyal\Copaiba #4 APRC RU181008M.qgd

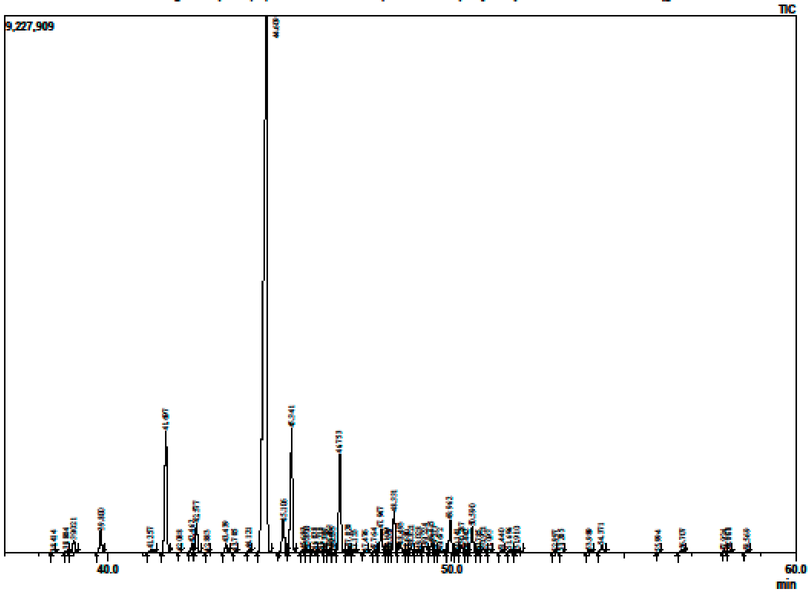

Comments:
PASS

Typical Ranges of Main Components:

Peak Report TIC

R.Time	Name	Area%
38.414	Bicyclo-<nonane>	0.03
38.804	Bicycloelemene	0.12
39.021	Elemene <delta->	0.81
39.800	Cubebene <alpha>	1.38
41.257	Ylangene<alpha->	0.23
41.697	Copaene <alpha>	7.86
42.088	Elemene<beta-cis>	0.02
42.462	Cubebene <beta>	0.58
42.577	Elemene<beta-trans>	1.88
42.883	Copaene <alpha>	0.02
43.439	Cyperene	0.55
43.705	Gurjunene <alpha->	0.04
44.121	Bergamotene<alpha-cis->	0.14
44.609	Caryophyllene <trans>	53.90
45.106	Elemene <gamma>	2.13
45.341	Bergamotene <trans-alpha>	7.75
45.683	Aromadendrene	0.18
45.800	Farnesene-Z-beta	0.24
46.028	Humulene<beta>	0.17
46.210	Santalene <beta-, epi->	0.12
46.349	Muurola-3,5-diene<cis->	0.07
46.463	Murrola-3,5-diene <trans>	0.34
46.555	Farnesene-<(E)-beta->	0.23
46.753	Humulene <alpha>	6.48
47.020	Alloaromadendrene	0.36
47.155	Muurola-4(14),5-diene <cis>	0.05
47.476	Elemene <isomer>	0.05
47.764	Cadina-1(6),4-diene<cis->	0.10
47.947	Cadina-1(6),4-diene <trans>	1.55
48.109	Curcumene <gamma>	0.10
48.207	Amorphene <alpha>	0.14
48.331	Germacrene D	2.61
48.493	Farnesene <alpha-cis-cis->	0.60
48.680	Selinene <delta>	0.05
48.821	Selinene <beta->	0.36
49.028	Muurola-4(14),5-diene <trans>	0.33
49.224	Bicyclogermacrene	0.60
49.415	Muurolene <alpha>	0.68
49.511	Bisabolene <cis-alpha->	0.17
49.672	Cadinene <beta->	0.20
49.962	Bisabolene <beta->	2.15
50.141	Bisabolene <Z-gamma>	0.17
50.287	Amorphene <delta>	0.53
50.442	Copaiba	0.07
50.590	Cadinene <delta->	1.72
50.785	Calamenene <trans>	0.08
50.921	Sesquiphellandrene <beta>	0.35
51.093	Bisabolene-<(E)-gamma->	0.04
51.440	Cadina-1,4-diene <trans->	0.08
51.696	Cadinene<alpha->	0.15
51.910	Bisabolene <trans-alpha>	0.27
52.997	Germacrene B	0.05
53.205	Calcorene <beta->	0.03
53.989	Caryophyllene alcohol	0.18
54.371	Caryophyllene oxide	0.50
55.994	Humulene epoxide II	0.03
56.707	Humulene-1,6-dien-3-ol	0.18
57.924	Muurolol <epi-alpha>	0.05
58.068	Cadinol <delta>	0.08
58.569	Cadinol<alpha->	0.08
		100.00

Figure S15. Copaiba essential oil CC3a

Sample Information

Analyzed by	: Dr. Prabodh Satyal
Analyzed	: 10/9/2018 9:35:25 AM
Sample Type	: Essential Oil
Sample Name	: Copaiba (#5)
ARC#	: RU181008N SAP# 00000
Injection Volume	: 0.10
Data File	: C:\Users\user\Desktop\QA Data File\psatyal\Copaiba #5 APRC RU181008N.qgd

Chromatogram Copaiba (#5) C:\Users\user\Desktop\QA Data File\psatyal\Copaiba #5 APRC RU181008N.qgd

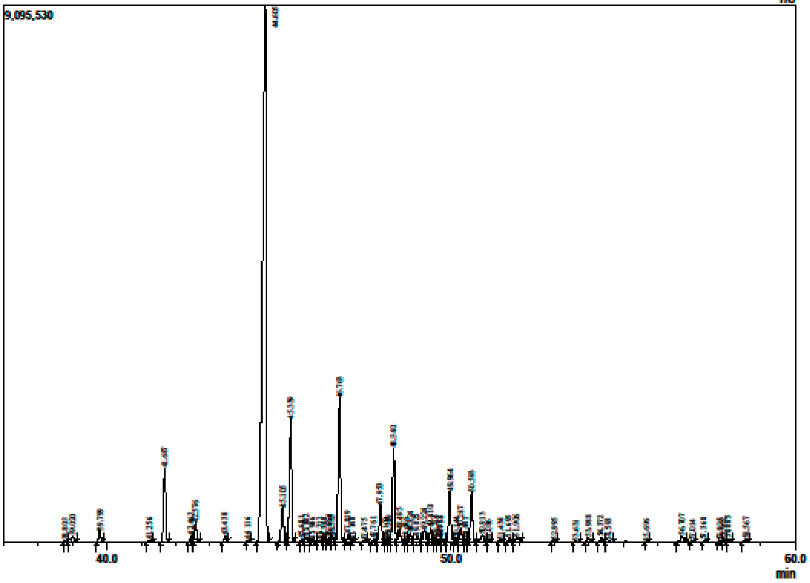

Comments:
PASS

Typical Ranges of Main Components:

Peak Report TIC

R.Time	Name	Area%
38.803	Bicylcoelemene	0.09
39.020	Elemene<delta->	0.34
39.799	Cubebene <alpha>	0.66
41.256	Ylangene<alpha->	0.10
41.687	Copaene <alpha>	4.26
42.462	Cubebene <beta>	0.39
42.576	Elemene <beta>	1.16
43.438	Cyperene	0.34
44.116	Bergamotene<alpha-cis->	0.11
44.605	Caryophyllene <trans>	48.84
45.105	Elemene <gamma>	2.15
45.339	Bergamotene <trans-alpha>	7.18
45.681	Aromadendrene	0.17
45.802	Farnesene-Z-beta	0.30
45.986	Humulene<beta->	0.17
46.212	Santalene <beta-, epi->	0.14
46.344	Murrola-3,5-diene <cis->	0.09
46.464	Murrola-3,5-diene <trans>	0.26
46.555	Farnesene<(E)-beta->	0.31
46.763	Humulene <alpha>	8.85
47.019	Alloaromadendrene	0.49
47.148	Muurola-4(14),5-diene <cis>	0.08
47.475	Elemene <isomer>	0.08
47.761	Cadina-1(6),4-diene <cis->	0.17
47.953	Cadina-1(6),4-diene <trans->	2.31
48.109	Curcumene<gamma>	0.14
48.209	Amorphene <alpha>	0.21
48.340	Germacrene D	5.56
48.495	Farnesene <alpha-cis-cis->	0.81
48.686	Selinene <delta>	0.10
48.824	Selinene <beta->	0.52
49.025	Muurola-4(14),5-diene <trans>	0.49
49.224	Bicyclogermacrene	0.98
49.410	Muurolene <alpha>	0.91
49.514	Bisabolene <cis-alpha->	0.26
49.672	Cadinene <beta->	0.22
49.735	Copaiba	0.11
49.964	Bisabolene <beta->	3.02
50.144	Bisabolene <Z-gamma>	0.26
50.287	Amorphene <delta>	0.81
50.441	Copaiba	0.11
50.593	Cadinene <delta->	2.90
50.913	Sesquiphellandrene <beta>	0.65
51.086	Bisabolene <E-gamma>	0.06
51.438	Cadina-1,4-diene <trans->	0.14
51.695	Cadinene<alpha->	0.24
51.906	Bisabolene <trans-alpha>	0.42
52.995	Germacrene B	0.15
53.631	Maaliol	0.07
53.988	Caryophyllene alcohol	0.32
54.372	Caryophyllene oxide	0.28
54.593	Globulol	0.06
55.696	Ledol	0.04
56.707	Humulane-1,6-dien-3-ol	0.46
57.014	Cubenol<1-epi->	0.05
57.360	Copaiba	0.06
57.825	Muurolol <alpha>	0.07
57.922	Muurolol <epi-alpha>	0.13
58.065	Cadinol <delta>	0.19
58.567	Cadinol<alpha->	0.19
		100.00

Figure S16. Copaiba essential oil CC3b

Supplemental Table S1. Summary of GC-MS data for mandarin essential oils

RT (min)	Name	CND (%)	CNC1 (%)	Literature (%, references)
12.673	α-Pinene	2.38	0.60	1.41-4.87 (Schipilliti *et al.*)
15.179	β-Pinene	1.89	0.12	1.07-2.60 (Schipilliti *et al.*)
15.813	Myrcene	1.99	1.75	1.37-2.01 (Schipilliti *et al.*)
18.066	p-Cymene	2.97	0.94	
18.488	Limonene	65.86	92.56	65.30-77.82 (Schipilliti *et al.*)
20.280	ɣ-Terpinene	19.43	1.55	13.13-23.36 (Schipilliti *et al.*)

Supplemental Table S2. Summary of GC-MS data for turmeric essential oils

RT (min)	Name	TD (%)	TC2 (%)	Literature (%, references)
16.904	α-Phellandrene	0.09	1.25	0.42 (Naz *et al.*);<br>0.1 (*Leela et al.)*
18.064	p-Cymene	0.07	1.29	
18.364	Limonene	0	1.44	
18.547	1,8-cineole	0.06	3.76	
48.124	ɣ-Curcumene	1.14	0.12	
48.337	α-Curcumene	2.66	6.40	
49.202	α-Zingiberene	4.42	5.72	
49.987	β-Bisabolene	0.92	1.71	
50.944	β-sesquiphellandrene	2.75	8.67	
54.119	AR-Turmerol	0	1.36	
58.712	α-Turmerone	7.37	0.26	18.35 (Naz *et al.*);<br>10 (Leela *et al.)*
59.157	β-Turmerone	23.56	27.75	
59.482	AR-Turmerone	27.31	13.30	25.33 (Naz *et al.*);<br>31.1 (Leela *et al.*)
60.984	E-ɣ-Atlantone	2.05	0	
61.209	Curlone	10.29	11.89	12.50 (Naz *et al.*);<br>10.6 (Leela *et al.)*
65.023	E-α-Atlantone	1.95	1.81	

Supplemental Table S3. Summary of GC-MS data for Melissa essential oils

RT (min)	Name	MD1 (%)	MD2 (%)	MC2a (%)	MC2b (%)
18.376	Limonene	2.30	1.54	1.21	0.53
18.769	Z-β-Ocimene	1.41	0.19	0.01	0.18
19.444	E-β-Ocimene	3.67	1.06	0.06	1.23
23.021	Linalool	0.51	0.95	2.52	1.08
26.659	Citronellal	5.86	5.44	2.82	2.08
31.610	Nerol	0.23	1.24	1.63	1.53
31.776	Citronellol	1.23	1.37	0	1.10
32.645	Neral	15.30	19.92	28.97	17.26
33.380	Geraniol	1.82	3.37	3.42	4.50
33.856	Methyl Citronellate	1.68	0.67	0	0.17
34.708	Geranial	23.53	28.43	35.33	25.54
38.131	Methyl Geranate	1.41	0.86	0	0.50
41.857	Geranyl acetate	0.68	2.53	4.41	5.20
44.566	β-Caryophyllene	12.30	12.89	12.44	17.82
48.398	Germacrene D	9.88	3.61	0	5.67
49.449	α-Muurolene	1.03	0.21	0	0.26
49.773	E,E-α-Farnesene	2.25	0.19	0	0.28
50.624	Δ-Cardinene	1.54	0.80	0	1.02

Supplemental Table S4. Summary of GC-MS data for copaiba essential oils

RT (min)	Name	CD1 (%)	CD2 (%)	CD3 (%)	CC3a (%)	CC3b (%)
39.025	Δ-Elemene	1.12	0.70	1.06	0.81	0.34
39.804	α-Cubebene	1.51	1.56	1.33	1.38	0.66
41.707	α-Copaene	7.19	7.43	5.97	7.86	4.26
42.581	β-Elemene	1.67	1.44	1.28	1.88	1.16
44.634	β-Caryophyllene	45.24	41.89	49.10	53.90	48.84
45.112	ɣ-Elemene	2.26	2.82	1.76	2.13	2.15
45.352	α-Bergamotene	7.19	8.51	8.30	7.75	7.18
46.770	α-Humulene	7.18	6.67	8.25	6.48	8.85
47.960	trans-Cardina-1(6),4-diene	2.03	2.67	1.98	1.55	2.31
48.349	Germacrene D	5.16	1.20	4.57	2.61	5.56
49.975	β-Bisabolene	3.47	4.89	2.52	2.15	3.02
50.602	Δ-Cadinene	2.78	3.88	2.30	1.72	2.90
54.376	β-Caryophyllene oxide	0.52	0.17	0.18	0.50	0.28

Supplemental Table S5. List of primary antibodies

Antibody	Cat. No.	Vendor
MEK1	07-641	Millipore (Billerica, MA)
MEK2	9125	Cell Signaling (Danvers, MA)
ERK1/2	040-474	Protein Simple (Santa Clara, CA)
Akt	8312	Santa Cruz Biotech (Dallas, TX)
4EBP1	9644	Cell Signaling
STAT1	14994	Cell Signaling
STAT3	4904	Cell Signaling
STAT6	5397	Cell Signaling
HSP70	4872	Cell Signaling

Supplemental Table S6. Assignment of pI values to protein isoforms

Protein isoforms	pI
MEK1	6.08
pMEK1	5.76
ppMEK1	5.66
pppMEK1	5.53
ppppMEK1	5.44
pppppMEK1	5.39
ppppppMEK1	5.25
MEK2	6.0
pMEK2	5.75
ppMEK2	5.63
pppMEK2	5.44
ppppMEK2	5.40
pppppMEK2	5.29
ERK2	6.63
pERK2	6.18-6.56
ppERK2	5.76
ERK1	5.95
pERK1	5.56
ppERK1	5.39
4EBP1	5.21
p4EBP1	5.06
pp4EBP1	4.60-4.99
AKT	6.05
pAKT	5.65
ppAKT	5.45
pppAKT	5.07-5.30
STAT3	5.84
pSTAT3	5.71
ppSTAT3	5.40-5.65
